# Interventions targeting working memory in 4–11 year olds within their everyday contexts: A systematic review

**DOI:** 10.1016/j.dr.2019.02.001

**Published:** 2019-06

**Authors:** Anita Rowe, Jill Titterington, Joni Holmes, Lucy Henry, Laurence Taggart

**Affiliations:** a Institute of Nursing and Health Research, Ulster University, Shore Road, Newtownabbey, Co Antrim BT37 0QB, Northern Ireland, United Kingdom; b MRC Cognition & Brain Sciences Unit, University of Cambridge, 15 Chaucer Road, Cambridge CB2 7EF, England, United Kingdom; c Division of Language and Communication Science, City, University of London, 10 Northampton Square, London EC1V 0HB, England, United Kingdom

**Keywords:** Working memory, Children, Near-transfer, Far-transfer, Durability

## Abstract

It has been suggested that diverse interventions applied within children’s everyday contexts have the potential to improve working memory (WM) and produce transfer to real-world skills but little is known about the effectiveness of these approaches. This review aims to examine systematically the effectiveness of non-computerised interventions with 4–11 year olds to identify: (i) their effects on WM; (ii) whether benefits extend to near- and far-transfer measures; (iii) if gains are sustained over time; (iv) the active ingredients; and (v) the optimum dosage. Searches were conducted across 12 electronic databases using consistent keywords. Papers were screened by title and abstract (*n =* 6212) and judged against pre-defined eligibility criteria (*n =* 63). Eighteen papers were included in the review. They used a range of non-computerised WM intervention approaches that included: (i) adapting the environment to reduce WM loads; (ii) direct WM training with and without strategy instruction; and (iii) training skills which may indirectly impact on WM (physical activity, phonological awareness, fantastical play and inhibition). Both direct training on WM tasks and practicing certain skills that may impact indirectly on WM (physical activity, fantastical play and inhibition) produced improvements on WM tasks, with some benefits for near-transfer activities. The common ingredient across effective interventions was the executive-loaded nature of the trained task i.e., training on a task that taps into attentional and processing resources under executive control and not just the storage of information. Few studies reported dosage effects, measured far-transfer effects (*n =* 4), or tested the durability of gains over time (*n =* 4). The lack of a clear theoretical framework in many of the included studies resulted in ambiguous predictions about training and transfer effects, and inadequate use of outcome measures. Methodological issues also constrain the strength of the evidence, including: small samples sizes; an absence of blinding of participant and outcome assessors; and lack of active control groups. Further well-designed and controlled studies with clear theoretical underpinnings are required to expand and enhance the evidence base. The heterogeneity of the interventions and of the study designs (randomised and non-randomised) in the included papers limited the synthesis of evidence across studies. However, this diversity enabled the identification of key ingredients, notably the training of executive-loaded WM tasks, which can help inform novel approaches to WM intervention in everyday contexts.

## Highlights

• Non-computerised training can improve working memory and near-transfer skills.
• Training other skills (physical activity, play, inhibition) is beneficial indirectly.
• Tapping into attentional resources (executive control) is key to task effectiveness.
• Further studies need clear theoretical underpinnings and rigorous methodology.
• Outcome measurement and dosage need greater consideration.

## Introduction

Speculation that diverse interventions applied within young children’s everyday contexts have the potential to improve their working memory (WM)^1^ skills and produce transfer to real-world skills such as attention, language and academic attainment has increased in recent years. However, such approaches have received limited attention to date and questions about their effectiveness remain unanswered. This review aims to systematically explore the effectiveness of such interventions and discuss whether they may provide an ecologically valid alternative to the widely adopted computerised WM training approach.

### What is working memory?

WM is the ability to hold in mind and mentally manipulate information over short periods in the face of distraction (Allen et al., 2009, Baddeley and Hitch, 1974, Cowan, 2008). The capacity of WM is limited (Broadbent, 1975, Cowan, 2001, Miller, 1956) meaning a finite amount of information can be held and processed in mind at any given time. WM limits the number and types of tasks we can carry out concurrently, because they are competing for the same limited cognitive resource (Henry, 2012). It develops more in the first 10 years of life than at any other point across the rest of the lifespan (Alloway & Alloway, 2015a) and reaches adult capacity levels around the age of 14 years (Gathercole, Pickering, Ambridge, & Wearing, 2004).

### The impact of working memory difficulties in the classroom

WM supports many everyday activities from reading to learning how to use a new device. It underpins many thinking processes (Henry, 2012), and is strongly linked with attention (Bunting and Cowan, 2005, Cowan et al., 2006), language learning (Baddeley et al., 1998, Weiland et al., 2014); mental arithmetic (Cragg, Richardson, Hubber, Keeble, & Gilmore, 2017); reading development (Kudo et al., 2015, Swanson et al., 2009); and sensory and motor skills (Alloway and Archibald, 2008, Leonard et al., 2015). Consequently, poor WM is associated with a wide range of learning difficulties, including specific language impairment^2^ (Archibald & Gathercole, 2007), dyslexia and reading difficulties (Jeffries and Everatt, 2003, Jeffries and Everatt, 2004, Swanson, 2003) and dyscalculia and mathematical learning problems (Geary et al., 2004, Szucs et al., 2013). Children with weak WM skills have difficulties coping with almost all classroom activities including: remembering and carrying out instructions (Engle et al., 1991, Jaroslawska et al., 2016, Jaroslawska et al., 2016); problem-solving (Swanson, Jerman, & Zheng, 2008); and planning, organising and keeping track of tasks (Alloway et al., 2009, Gathercole et al., 2006). Teachers frequently describe such children as inattentive and distractible (Alloway et al., 2009). Children with poor WM struggle to cope with the heavy WM loads of the classroom, leading them to fail to complete individual learning activities. Over time, these failures result in poor educational progress, as problems accumulate and affect knowledge across classroom activities. This may explain why WM is one of the best single predictors of a child’s academic achievement (Alloway and Alloway, 2015a, Alloway et al., 2009, Gathercole and Pickering, 2000, Gathercole et al., 2016).

### Models of working memory

There are several theoretical models and accounts of the structure and function of WM. Componential theories assume the WM system consists of separate stores for the maintenance of different types of information and for executive control (Baddeley & Hitch, 1974). Baddeley and Hitch (1974) multicomponent model of WM (and its subsequent revisions, e.g., Baddeley, 2000) has received considerable empirical support and been highly influential over the past 40 years. It has been particularly valuable in advancing our understanding of how children learn and acquire language (e.g. Archibald, 2017, Baddeley et al., 1998, Gathercole and Baddeley, 1993, Holmes and Adams, 2006, Swanson, 1994, Swanson, 2017), which is why it has been used to underpin much of the discussion in this systematic review. Other theories, such as that proposed by Cowan, 2005, Cowan, 2008, are based on an assumption that WM is an activated portion of long-term memory (LTM) rather than a distinct entity, and therefore its capacity is related to attentional resources i.e., the current contents of WM are activated representations of LTM.

Although these models have obvious differences, they agree that WM involves two key features: high-level attentional control plus temporary storage. According to most theories, memory tasks can be differentiated by: (a) simple span tasks that involve the temporary, passive storage of material; and (b) complex span tasks that require the concurrent storage and processing of information and rely on attentional resources under executive control. The fractionation of simple and complex tasks is supported by evidence that complex memory span measures are more strongly associated with other higher-order cognitive (executive) functions and language skills than simple memory span tasks (Engle, 2002, Gathercole et al., 2004, Henry and Botting, 2017). The relationship between complex span performance and other cognitive and learning abilities suggests that improving executive aspects of WM might lead to generalised gains across a wide range of skills.

### Research into the effectiveness of WM training

Research into the effectiveness of WM training has stemmed from two (sometimes conflicting) perspectives (Wass, Scerif, & Johnston, 2012). From a theoretical perspective, the goal has been to understand underlying cognitive processes by exploring the extent to which training can improve WM and identifying variables that moderate or mediate the effects of training and transfer to other cognitive functions (Gathercole et al., 2019, Jaeggi and Buschkuehl, 2014, Schwaighofer et al., 2015). From an applied perspective, the therapeutic value of WM training lies in its potential to ameliorate problems associated with poor WM. The focus of applied research has been on investigating the impact of WM training on outcomes relating to real-world skills such as attention, language and academic attainment. The benefits of practice on the trained activities should produce improvements both on untrained WM tasks (near-transfer) and on measures of other abilities known to depend on WM, such as educational outcomes and attention (far-transfer).

Morra and Borella (2015) suggest that WM research has evolved in three waves. The first was fuelled by cumulative evidence that WM may not be the fixed entity it was once thought to be (e.g., Ericsson and Chase, 1982, Klingberg et al., 2002) but could be altered via training. Most of this evidence was generated through a large number of quasi-experimental studies of the effects of computerised WM training, the majority of which used the Cogmed Working Memory Training program (Cogmed, 2005). Perhaps inevitably, this was followed by a second wave in which questions were raised about the methodological quality of the studies (e.g. lack of randomised controlled trials, failure to blind participants to condition and the absence of adequate control groups – see Shipstead, Hicks, and Engle (2012) for a comprehensive review). Theoretical questions were also asked about why or how repeated practice on tasks would improve WM capacity (Janneke et al., 2016, Melby-Lervåg and Hulme, 2013, Melby-Lervåg et al., 2016, Morrison and Chein, 2012, Sala and Gobet, 2017, Shipstead et al., 2012). The most rigorous studies, meta-analyses and systematic reviews provide evidence that computerised training leads to gains on the trained tasks and on untrained activities when the untrained tasks have many features in common with the trained tasks (e.g. Barnett and Ceci, 2002, Gathercole et al., 2019, Noack et al., 2009, Soveri et al., 2017, Sprenger et al., 2013, Von Bastian and Oberauer, 2014). Some meta-analyses provide evidence for near-transfer effects, but far-transfer effects are often small and not sustained over time (Schwaighofer et al., 2015). Taken as a whole, transfer to WM tasks with few overlapping features with the trained activities is inconsistent (see Gathercole et al., 2019 for a review).

There has been considerable debate in the field of WM research about: (i) the reasons for inconsistent near-transfer effects following computerised WM training; (ii) the lack of and unsustainability of any far-transfer effects; (iii) the factors that may mediate and moderate training and transfer effects; and (iv) the potential theoretical mechanisms underpinning these findings (e.g., Gathercole et al., 2019). Questions have been asked about the impact of experimental factors (e.g., use of active control conditions, location and levels of supervision, instructions and feedback, and methods of statistical analysis). The nature of the learning involved has also been questioned (e.g., whether paradigms support the development of new cognitive routines), and the influence of individual differences in learner characteristics are increasingly investigated (e.g., age, motivation and pre-existing abilities) (Jaeggi et al., 2012, Morra and Borella, 2015). However, there is increasing evidence that near-transfer to structurally dissimilar WM tasks is elusive and that the role of individual differences in mediating these is negligible. Recent studies, taking a Bayesian approach to statistical analysis to explicitly investigate these questions in young and older adults, found an absence of near and far-transfer effects following computerised WM training, which could not be explained by individual differences between participants (De Simoni and von Bastian, 2018, Guye and von Bastian, 2017).

The third wave of WM research questions the utility of intervention approaches that cannot account for how the underlying capacity of WM is altered by training (from a theoretical perspective) and do not improve outcomes on real-world skills (from an applied perspective). In particular, the lack of consistent evidence for far-transfer effects from computerised WM training (Dunning et al., 2013, Holmes et al., 2015, Shipstead et al., 2012, Simons et al., 2016) has underscored the need for alternative intervention approaches. On the basis that gains transfer to activities with overlapping features to the trained tasks, there have been calls for training to be embedded within the typical activities in children’s everyday contexts in which benefits are needed (e.g. educational activities that depend on WM) (Dunning and Holmes, 2014, Dunning et al., 2013).

Taking WM training from the experimental setting into everyday contexts has several disadvantages. When interventions are applied in real-life settings (particularly groups), it is harder to control the training regime including the quality, dose and fidelity of its delivery (Jaeggi & Buschkuehl, 2014). Extracting individual child performance is also challenging. However, the variation in training tasks and materials afforded through this approach may be more motivating than computerised training for young children (Wass et al., 2012) and reduce the frustration they feel as tasks increase in difficulty (Jaeggi et al., 2012). It has been speculated that a diverse range of activities may impact on WM (Diamond, 2012, Otero et al., 2014) but little is known about their effectiveness.

### Rationale and scope of this review

The current review is, to our knowledge, the first to focus specifically on the effectiveness of non-computerised WM interventions applied within children’s everyday contexts. It is a novel investigation of intervention effects on trained WM skills, on untrained but similar WM skills (near-transfer effects) and on other untrained, real world skills (far-transfer effects). The review seeks to contribute to the third wave of WM research by mapping the types of interventions that have been implemented with young children in everyday contexts and examining the theoretical framework/s used to underpin them (Morra & Borella, 2015). This recognises the need to consider *why* and *how* interventions are intended to improve WM function. Thus, the questions posed in this review are consistent with a realist research perspective (Fletcher et al., 2016, Wong et al., 2014). This goes beyond asking ‘what works’, to ask ‘for whom it works and under what circumstances’ (Pawson & Tilly, 1997, p. 72) and recognises that all interventions, whether explicitly stated or not, are based on causal assumptions (Moore & Evans, 2017). The review includes an examination of intervention intensity (dosage) and which intervention tasks (if any) appear to be the active ingredients i.e., those attributes of the treatment that play a role in its effects (Hart et al., 2014).

In the case of WM interventions, identifying active ingredients highlights the need to spell out what memory measures really assess and the skills targeted in training (Morra & Borella, 2015). It is evident that this has not always occurred, or at least that it has not been clearly articulated. Indeed, the terminology used to describe WM tasks is inconsistent in the literature and the differentiation between simple span and complex span tasks is often unclear. Short-term memory typically describes simple span tasks (storage-only). Working memory is an umbrella term describing all memory tasks (simple span and complex span tasks), but can also be used to describe only complex tasks (storage plus processing).

To avoid confusion, clear operational definitions are essential. Here, the term executive-loaded working memory (ELWM) (Henry, 2012) is used to refer to tasks with an attentional or processing load (e.g. complex span tasks). Short-term memory (STM) describes storage only tasks (simple span tasks) and working memory (WM) is used as an umbrella term to refer to all memory tasks. Tasks are also defined by modality i.e., verbal or visuospatial. Thus four aspects of WM are delineated (verbal and visuospatial STM and ELWM) to categorise trained tasks and outcome measures. Table 1 provides examples of tasks representing each aspect of WM.

**Table 1:** Task examples for each aspect of WM.

Working memory (WM)			
Short-term memory (STM) Tasks: simple span (storage only)		Executive-loaded working memory (ELWM) Tasks: complex span (storage plus processing)	
Verbal (VSTM)	Visuospatial (VSSTM)	Verbal (VELWM)	Visuospatial (VSELWM)
Digit span Word span Non-word span	Dot matrix Corsi block tapping	Backward digit span Listening span	Backward Corsi block tapping Odd one out span

## Aim

The aim is to examine systematically the effectiveness of non-computerised interventions targeting WM in 4–11 year olds applied within their everyday contexts.

### Research questions

1. What types of WM interventions are implemented and what is their theoretical underpinning?
2. What are the effects of the interventions on WM, and which aspects of WM (if any) are impacted?
3. Do WM gains made (if any) extend to:
  (a) Similar untrained WM tasks (near-transfer effects)?
  (b) Dissimilar abilities linked with WM i.e., real world skills including language, literacy, numeracy and paying attention in class (far-transfer effects)?
4. Are WM gains durable over time?
5. What are the active ingredients and the optimum dosage of effective interventions?

## Methods

### Systematic review protocol

The protocol for this review was reported in line with the PRISMA-P (2015) (Moher et al., 2015) checklist. It was registered with and published by the International Prospective Register of Systematic Reviews (PROSPERO) which is available at https://www.crd.york.ac.uk/PROSPERO/ (Registration number CRD42017056360).

### Eligibility criteria

The PICO model (population, intervention, comparison, and outcomes) (Booth & Fry-Smith, 2004) was used in the development of the eligibility criteria for the inclusion of studies.

*Population: Studies must have been conducted with children aged 4*–*11 years.* This represents the age span of children attending mainstream primary schools in the United Kingdom (UK). This population is of interest to the review team due to suggestions that WM interventions applied in everyday contexts may be more ecologically valid and motivating for younger children than computerised training (Jaeggi et al., 2012, Wass et al., 2012). Where participants in a study spanned the age band (4–11 years) but also included participants on either side of this range e.g., 7–14 year olds, the age of the majority of participants was considered. Papers were included if more than 50% of the participants were aged 4–11 years. In the UK, the mainstream school population can include typically developing children and those with diagnosed or undiagnosed developmental difficulties or intellectual disability. Studies were therefore not included or excluded on this basis.

*Intervention: Studies may have implemented any intervention that targets WM and is applied within children’s everyday contexts.* Computerised WM training studies were excluded from the current review as these have been rigorously reviewed and debated previously. Interventions may have been delivered by a teacher, healthcare professional or researcher and may have been carried out in a school, clinical or research setting, but must have provided a total duration of more than one session and less than one year of intervention.

*Comparison: Studies must have a randomised controlled, quasi-experimental or single case experimental design.* While adhering to the core principles of systematic review methodology in the transparent reporting of study selection, analysis and synthesis, this review applies realist principles and judiciously includes non-randomised studies (Gough et al., 2012, Pawson, 2006). This is consistent with: previous systematic reviews/meta-analyses of the topic e.g., Melby-Lervåg and Hulme, 2013, Melby-Lervåg et al., 2016, Danielsson et al., 2015 and the early stage of development of much of the research in this field whereby even weaker studies may provide information of value (Petticrew, 2015). The risk of bias that this may introduce is considered and reported transparently.

*Outcomes: Studies must have at least one pre- and post-intervention measure of WM. Studies that investigated training on other tasks that may indirectly impact on WM must also include at least one measure of an aspect of the trained task (s).* The primary outcome of interest is the effect on WM. In order to discern whether the effects on WM can be attributed to the intervention, the trained task must have been objectively measured (Melby-Lervåg and Hulme, 2013, Melby-Lervåg et al., 2016). Studies that explicitly taught cognitive strategies to enhance direct WM training were included regardless of whether they had measured children’s use of the strategies because the use of strategies is seen as an integral part of the WM intervention. Near- and far-transfer effects and the durability of effects over time are secondary outcomes, so studies were included regardless of whether these had been measured. Interventions may have the potential to demonstrate such effects even if these have not been measured yet. The current review will contribute to ongoing debates about transfer effects and the time-frame within which they may be demonstrable (Redick, Shipstead, Wiemers, Melby-Lervåg, & Hulme, 2015).

### Data sources

The search strategy (outlined below) was applied to twelve electronic databases: MEDLINE (Ovid) Pubmed; EMBASE; CINAHL Plus; AMED; PsycINFO; Scopus; Educational Resource Information Center (ERIC); Web of Science; British Education Index; Cochrane Central Register of Controlled Trials (CENTRAL); Linguistics and Language Behavior Abstracts (LLBA). The authors also inspected the reference lists of relevant papers. Where papers could not be directly accessed via these databases authors were contacted directly. Searches imposed a date limit with studies pre-1974 (when Baddeley and Hitch first proposed the multi-component model) being excluded. Due to resource constraints, searches were restricted to studies in the English language.

### Search strategy

The search strategy was devised through scoping the literature for keywords indexed on published papers, reflection on search terms used in previous reviews e.g., Melby-Lervåg and Hulme (2013), and consultation with the subject-specific librarian at Ulster University. Following trials of two draft search strategies (January 2017), final searches were conducted (February 2017) with Boolean phrases using the terms (“working memory” OR “short-term memory” OR “executive function*”) AND child* AND (training OR intervention*OR treatment OR therap* OR program*). Searches were updated on a regular basis throughout the review and just prior to its completion (July 2017). The results were exported to Refworks (2.0) and duplicates were removed. An excel spreadsheet was then created to manage the bibliography of all potential papers.

### Study selection

As anticipated, electronic database searches produced a large number of results (*n =* 6262). This was considered unavoidable as narrowing the search terms would have excluded relevant studies. The first author screened the results by title and removed studies which clearly did not meet the criteria (*n =* 5903). For example, titles which explicitly stated that the intervention implemented was computerised or that it was conducted with adults were immediately excluded. Where there was any doubt about the eligibility of a study, it was included for further screening. The abstracts of the remaining studies (*n =* 359) were screened again by the first author and this process was checked by the second author. Abstracts frequently provided insufficient detail to support inclusion/exclusion from the review and, where there was any uncertainty, the full paper was considered. Following this, all potential papers were reviewed against a checklist of the eligibility criteria (*n =* 69) and 50% of these were reviewed independently by two other members of the review team. The first reviewer compiled a table to aggregate the information on each reviewer’s decision, which was discussed at a face-to-face meeting of the review team in May 2017 where there was full agreement.

### Data extraction

The data extraction process was guided by the PICO framework (Booth & Fry-Smith, 2004). Population, comparison and outcomes data were extracted from each study and collated on an excel spreadsheet. Data relating to the theoretical underpinning, content and delivery (including dosage) of each intervention were extracted using the Template for Intervention Description and Replication (TIDieR) Checklist (Hoffmann et al., 2014). When available, data on four variables of intervention dosage were extracted following definitions proposed by Warren, Fey, and Yoder (2007): (i) dose - the number of properly administrated trials per training session; (ii) dose frequency – the number of times the dose is provided per day or per week (which may also include session duration); (iii) the total intervention duration – the time period over which the intervention is presented e.g., 3 weeks or 6 months (see Warren et al., 2007) and iv) cumulative intervention intensity i.e., dose × dose frequency × total intervention duration (e.g., 10 trials × 4-times per week × 12 weeks = 480).

### Data analysis

After careful consideration, it was decided that a meta-analysis was not appropriate for this review. This decision was partially based on the purpose of the review and the research questions posed. The current review aims to map a diverse range of studies to investigate the best approach to WM intervention for young children. The Cochrane Handbook for Systematic Reviews advises that meta-analyses are not indicated for reviews with this focus (Deeks, Higgins, & Altman, 2008). Further, a meta-analysis could be misleading, given the clinical heterogeneity of the included interventions (Hoffman, 2015). The interventions are diverse in their approach, content, dosage and outcomes measured so the interpretation of effects in a meta-analysis would be challenging. The low number of studies in each intervention category would also result in very low power for any sub-group meta-analyses so it would be difficult to make generalisations from the results (Borenstein, Hedges, Higgins, & Rothstein, 2009)

The data analysis process involved three steps. First, the included studies were categorised according to the type of WM intervention implemented (see results section). Next, the results from each study were examined in respect of: the primary outcomes - effects on aspects of WM (research objective 2); and the secondary outcomes- near- and far-transfer effects and the durability of intervention effects over time (research objectives 3 and 4). During this process, all of the trained WM tasks and outcomes were defined according to which of the four aspects of WM they involved because this was not always clearly identified in the papers. In the majority of cases, studies did not clearly differentiate between trained and untrained but similar tasks (near-transfer effects) when reporting outcomes. To be deemed effective, a logical pattern of treatment effects must have been demonstrated. Where an intervention trained other skills to indirectly impact WM, there must have been improvements on the trained task as well as WM (Melby-Lervåg and Hulme, 2013, Melby-Lervåg et al., 2016, Redick et al., 2015). Likewise, interventions were considered to have produced far-transfer effects, if near-transfer effects were observed (Redick et al., 2015). The third step involved extracting the results from those studies which demonstrated positive treatment effects and synthesizing the evidence for the overall effectiveness of the interventions on the primary and the secondary outcomes. This enabled consideration of the active ingredients and optimum dosage implemented in the most effective interventions (research objective 5).

### Assessing risk of bias and confidence in cumulative evidence

The external and internal validity of the studies was investigated using the Cochrane Collaboration tool for assessing the risk of bias (Higgins & Altman, 2008) which can be applied to both randomised and non-randomised studies (Reeves, Deeks, Higgins, & Wells, 2008). When considering the strength of the evidence, the criteria suggested by Redick et al. (2015) for the evaluation of WM training effectiveness were also considered, prompting particular attention to the nature of the control condition. Reporting quality was measured using the CONSORT guidance on the reporting of randomised trials (Schulz, Altman, Moher, & for the CONSORT Group, 2010) and the TREND statement (Des Jalais, Lyles, Crepaz, & the TREND Group, 2004) for non-randomised study designs. The Grading of Recommendations Assessment, Development and Evaluation working group methodology (GRADE) (GRADE working group, 2004), increasingly recommended for systematic reviews (Shamseer et al., 2015), was used to assess the cumulative strength of the evidence for the synthesized findings on the primary and secondary outcomes.

## Results

### Study selection and characteristics

**Fig. 1:**
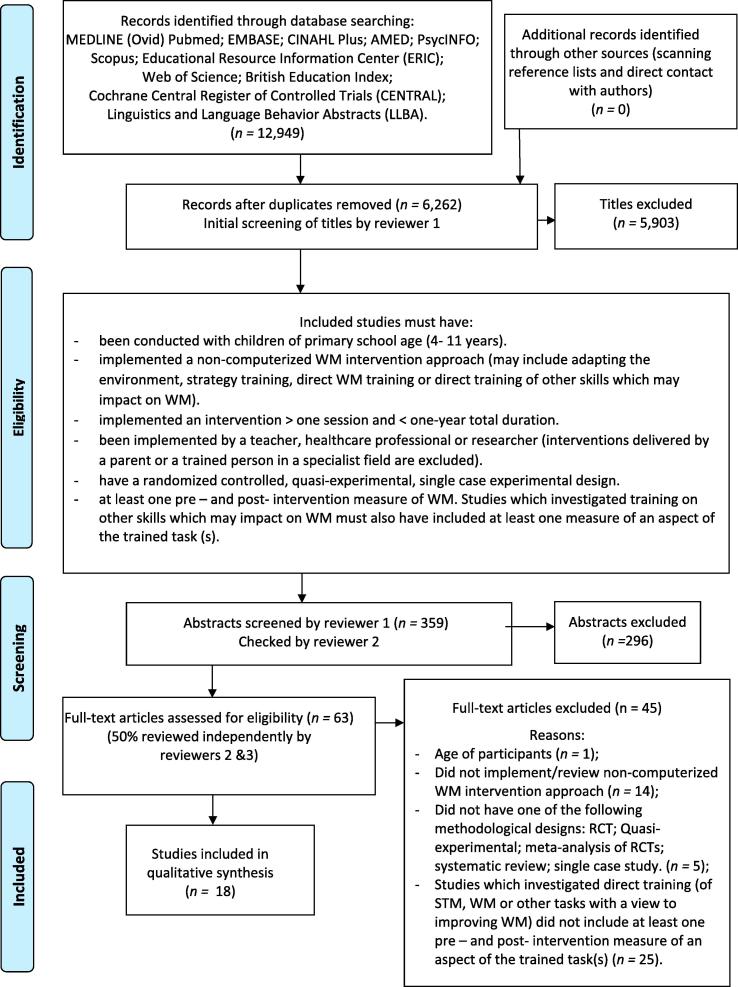
Flow Diagram for search strategy with criteria and reasons for exclusion following the PRISMA statement (Moher, Liberati, Tetzlaff, Altman, & The PRISMA Group, 2009).

The number of studies screened at each stage of the selection process and the reasons for exclusion are shown in Fig. 1. Most of the excluded studies (*n =* 25) were omitted on the basis of the outcomes measured. Eighteen studies have been included in the review and an overview of their characteristics is provided in Table 2. The studies were carried out in eight countries, with most being conducted in the United States (*n =* 5), Italy (*n =* 5) and the UK (*n =* 3). Publication dates ranged from 1994 to 2016 and seventeen were published after the publication of the CONSORT (Schulz et al., 2010) and TREND (Des Jalais et al., 2004) statements. The majority of studies (*n =* 12) were conducted with typically developing children, only one study focused on children with WM difficulties and none specifically targeted children from low socio-economic backgrounds. To be included in the review, studies must have been conducted with children aged 4–11 years. In the final study selection, participants ranged from 4 to 10 years (mean age = 7.8 years). Fewer than 50% of the studies (*n =* 7) were randomised controlled trials, only two studies used an active control group and none reported having undertaken a power calculation in determining sample size. In terms of outcome measurement, fewer than 50% of the studies (*n =* 7) measured more than one aspect of WM, and the use of standardised WM measures was limited. Verbal skills were more frequently measured than visuospatial WM. Few studies measured transfer effects, but more assessed far- (*n =* 7) than near-transfer effects (*n =* 4), with only one study explicitly investigating near-transfer effects using standardised WM assessments (Henry, Messer, & Nash, 2014). Appendix A provides the dosage data extracted from each paper, showing significant gaps in the reporting of treatment intensity.

**Table 2:** Summary of characteristics of all studies included in the review (*n =* 18).

PICO variable	Study characteristics
Population	Age range: 4; 4–10 years. Mean $\left(\bar{x}=7.8\;y\right)$ Status: Typically developing children (*n =* 12) Language impaired children (*n =* 1) Children with reading difficulties (*n =* 1) Children with identified WM difficulties (*n =* 1) Children considered at risk of learning difficulties (*n =* 1) Children with Down’s syndrome (*n =* 1) Overweight children (*n =* 1) Studies specifically targeting children from low socio-economic status (SES) (*n =* 0)
Intervention	Intervention approach: Adapting the environment (*n =* 1); Direct WM training without strategy instruction (*n =* 3) Direct WM training with strategy instruction (*n =* 5); Training skills that indirectly impact on WM (*n =* 9). – physical activity (*n =* 5); – phonological awareness skills (*n =* 2); – fantastical play (*n =* 1); – inhibition (*n =* 1). Where the intervention was delivered: via Skype (*n =* 1) school (*n =* 12) preschool setting (*n =* 1) gym (*n =* 1) No detail given (*n =* 3) Unit of delivery (grouping of participants): Individual (*n =* 8) Small group (*n =* 6) Whole class (*n =* 2) Combination of individual and small group (*n =* 2). Dosage: Total duration of intervention ranged from 10 days to 11 months Average total intervention duration in weeks $\left(\bar{x}=13\right)$
Comparison	Study design: Randomised controlled trials (*n =* 8) Quasi-experimental designs (*n =* 9) Case series (*n =* 1) Control group conditions: Active control groups (*n =* 2) No intervention control groups (*n =* 14) Own matched controls (*n =* 2) Sampling: Power analysis reported (*n =* 0) Sample size range across all included studies (*n =* 4 to *n =* 256). Average sample size $\left(\bar{x}=74\right)$
Outcomes	Measurement of WM: Verbal STM (*n =* 11) Visuospatial STM (*n =* 7) Verbal ELWM (*n =* 14) Visuospatial ELWM (*n =* 4) Studies which measured more than one aspect of WM (*n =* 7) Studies which implemented a standardised measure of WM (*n =* 5) Measurement of transfer effects: Near-transfer (*n =* 4) Far-transfer (*n =* 7) Durability over time (*n =* 4)

The first research objective was to identify the types of WM interventions implemented with 4–11 year olds in their everyday contexts and their theoretical underpinnings (the causal assumptions embedded within them). There is no agreed system for the classification of WM interventions that are applied in children’s everyday contexts (see Diamond and Lee, 2011, Otero et al., 2014 for examples). In this review, the included studies have been classified into four intervention types:

(1) Adapting the classroom environment: Only one study reviewed here investigated the effectiveness of this approach which does not require direct intervention with children. Instead, teachers are educated about how to detect children with WM difficulties and trained in how to adapt learning activities to reduce WM loads for these children. By reducing WM demands in the classroom, children with poor WM may successfully complete more learning tasks and consequently acquire knowledge across lessons.
(2) Direct WM training without strategy instruction: Three interventions involved repeated practice on verbal or visuospatial tasks requiring STM (storage only tasks) or ELWM (storage plus an additional attentional or processing load).
(3) Direct WM training with strategy instruction: Five studies investigated the effectiveness of explicitly teaching children to use cognitive strategies while practising WM tasks. The use of strategies to support the efficiency of WM emerges during childhood e.g., verbal rehearsal (repeating the to-be-remembered information out loud or in your head) develops at about 7 years (Gathercole, 1998). Other strategies, such as organization and grouping (Bjorkland & Douglas, 1997) and chunking information (Ottem, Lian, & Karlsen, 2007) develop later than this. It has been suggested that younger children may benefit more from this strategy-supported direct training than older children who may have established these skills already (St. Clair-Thompson and Holmes, 2008, St. Clair-Thompson et al., 2010).
(4) Training skills that indirectly impact on WM: A wide range of activities including martial arts, physical exercise, yoga, mindfulness, music, sports and traditional childhood games can impact on executive function skills including WM (Diamond, 2012, Otero et al., 2014). Other approaches including sensory-based interventions (Worthen, 2010), proprioceptively demanding exercise (Alloway & Alloway, 2015b) and motor skills training (Halperin et al., 2015) have also been suggested as benefitting WM. Nine studies were included that implemented a range of interventions: physical activity (*n =* 5); phonological awareness (*n =* 2); fantastical play (*n =* 1) and inhibition^3^ (*n =* 1). Studies investigating sensory-based and mindfulness interventions did not meet the eligibility criteria for this review.

Table 3 provides the theoretical underpinnings stated in the included papers and the methodological characteristics of each study. This shows that many of the studies lacked a clear theoretical account of why the intervention should impact WM. In particular, the studies looking at indirect training of WM through physical activity, fantastical play (Thibodeau, Gilpin, Brown, & Meyer, 2016), or inhibition (Volckaert & Noël, 2015) did not refer to theoretical models of WM in their study rationale.

**Table 3:** Characteristics of individual studies included in review (*n =* 18).

Intervention type	Study author and year	Population			Intervention type	Theoretical underpinning provided by study authors	Design	Control condition
		Status	Age in years (y)/months (m) E (C)	Total sample size				
Adapting the environment	Elliott et al. (2010)	TD	5–6 year olds 9–10 year olds	256	Adapting the environment Teachers divided into 2 groups and provided with training on: (1) WM intervention: the use of strategies to modify and reduce WM loads in the classroom; or (2) Behavioural teaching. the use of behavioural approaches in the teaching of directly targeted academic skills.	Baddeley and Hitch Working Memory Model. Based on hypothesis that reducing WM overload may result in improved learning.	Quasi-experimental with nested design. Two age groups participated in each of two successive cohorts.	No intervention
Direct WM training without strategy instruction	Banales et al. (2015)	R + WM	9.92y.	4	Direct WM without strategy instruction	Baddeley and Hitch Working Memory Model	Case series	Each child completed 8 weeks of verbal working memory training and 8 weeks of reading training, acting as their own matched control.
	Henry et al. (2014)	TD	7 year olds 84.2 m. (84.0 m.)	36	Direct WM without strategy instruction	Baddeley and Hitch Working Memory Model Training explicitly targets ELWM skills.	RCT	Active control: similar task with no memory element.
	Passolunghi and Costa (2016)	TD	5 year olds Group 1 65.8 m. Group 2 64.67 m. (64.4 m)	48	Direct WM without strategy instruction 2 trained groups: (1) WM training (2) numeracy training	Baddeley and Hitch Working Memory Model	RCT	No intervention
Direct WM training with strategy instruction	Caviola et al. (2009)	TD	“Fourth grade” 8–9 year olds	46	Direct WM training with strategy instruction	Reference to meta-cognitive literature and Baddeley and Hitch Working Memory Model but trained tasks not clearly differentiated as STM or ELWM.	Quasi-experimental	General cognitive strategies
	Comblain (1994)	DS	8 year olds	8	Direct WM training with strategy instruction	No theory provided	Quasi-experimental	No intervention
	Cornoldi et al. (2015)	TD	8–10 year olds	135	Direct WM training with strategy instruction	Baddeley and Hitch Working Memory Model Hypothesis that combining WM training with meta-cognitive strategies might promote more robust and persistent effects on problem solving.	Quasi-experimental with a cross-over design (2 groups taking part in 8 weeks of training consecutively).	When not in a training period, children took part in their usual mathematics lessons.
	Peng and Fuchs (2015)	LD	Strategy − 7.19y No-strategy 7.09y. Control 7.13y.	58	Direct WM with strategy instruction 2 intervention conditions: (1) WM with strategy (2) WM without strategy	The authors distinguish between WM tasks pertaining to either the verbal or visuospatial domain and refer to the debate between domain-general and domain- specific WM theories, hypothesising that domain-specific training will produce effects on related academic skills. They refer to 'Strategy Mediation Theory' - view that WM is a finite fixed capacity and performance is determined by the efficiency with which this capacity is used.	RCT	No intervention
	Witt (2011)	TD	9 year olds 116.13 m.	38	Direct WM training with strategy instruction	Baddeley and Hitch Working Memory Model. Training explicitly targets ELWM skills.	Quasi-experimental	No intervention
Training skills which may indirectly impact on WM - physical activity	Alesi et al. (2016)	TD	8.78y. (9.25y.)	44	Physical activity Football exercise program	No reference to WM theory. Based on concept that new tasks stimulate co-activation of prefrontal cortex and may impact on cognition.	Quasi-experimental	No intervention (“sedentary children”)
	Davis et al. (2007)	O	9.2y.	94	Physical activity 2 intervention conditions (1) low dose (20 min) (2) high dose (40 min)	No reference to WM theory.Based on concept that children’s cognitive functioning may be particularly sensitive to the influence of physical activity.	RCT	No-exercise control condition
	Kamijo et al. (2011)	TD	8.9y. (9.1y.)	43	Physical activity	No reference to WM theory. Based on evidence of a positive relation between cardiorespiratory fitness and neurocognitive functioning in children Hypothesised that greater improvements would be made on tasks with greater WM demands due to improvements in cognitive control.	RCT	Waitlist control
	Koutsandréou et al. (2016)	TD	CE group 9.3y. ME group 9.4y. (9.3y.)	71	Physical activity 2 trained groups: (1) Cardiovascular exercise program (CE group) Motor-demanding exercise program (ME group).	No reference to WM theory. Based on evidence of a motor-cognition link: that motor exercise leads to adaptation of brain areas, which might benefit cognition more than mere cardiovascular exercise.	RCT	Control group: assisted homework sessions
	Van der Niet et al. (2016)	TD	8.8y (8.9y)	112	Physical activity	No reference to WM theory. Based on evidence of the relationship between regular aerobic exercise and executive functions and suggestions that: exercise induces neurochemical and morphological changes in the brain areas associated with executive functioning; and that physical activities in which specific goals have to be achieved are cognitively demanding.	Quasi-experimental	Normal daily school routine (included 2 PE lessons per week)
Training skills which may indirectly impact on WM - phonological awareness	Melby-Lervåg and Hulme (2010)	TD	7y1m.	160	Phonological awareness 3 intervention conditions (1) phoneme awareness training (2) Rhyme training (3) vocabulary training.	Baddeley and Hitch Working Memory model.The authors aimed to Investigate the interrelationship between verbal memory and language processing, hypothesizing that phoneme training would enhance serial recall more than rhyme training based on the assumption that serial recall serial recall depends on the quality of the phonological representations of the words to be recalled (phonological representation hypothesis).	Quasi-experimental	No-intervention
	Van Kleeck et al., 2006, Van Kleeck et al., 1998	SLI	4–5 years Group 1 48.87 m. Group 2 60 m (71.5 m).	24	Phonological awareness	Baddeley and Hitch Working Memory model. Based on hypothesis that phonological awareness training improves the functioning of the phonological loop.	Quasi-experimental	1998 paper: No-intervention control
Training skills which may indirectly impact on WM - fantastical play	Thibodeau et al. (2016)	TD	3–5 years Group 1 50.62 m. Group 2 54.06 m. (52.37 m.)	110	Fantastical play 2 intervention conditions: (1) fantastical play (2) non-imaginative play	No reference to WM theory. Vygotsky’s theory that complex pretend-play provides natural experience in which cognitive skills are developed and that in fantastical play children may use WM to remember the rules and scripts in the pretence.	RCT	No intervention (business as usual)
Training skills which may indirectly impact on WM - inhibition	Volckaert and Noël (2015)	TD	4–5 years 60.13 m. (60.52 m.)	112	Inhibition	No reference to WM theory. Based on associations between executive functions and children’s externalising behaviours in pre-schoolers.	RCT	“Passive” control group -handicraft lessons

*Key:* C = control group (s); E = experimental group; TD = typically developing; DS = Down’s syndrome; SLI = specific language impairment; O = overweight; LD = at risk of learning difficulties; RD = reading difficulties’ R + WM = reading and WM difficulties. RCT: Randomised Controlled Trial.

### Results of individual studies

The main findings of each study and the evidence for each of the four identified intervention approaches are summarised below (see supplementary material Table A for a comprehensive overview).

1. Adapting the environment

One paper (Elliott, Gathercole, Alloway, Holmes, & Kirkwood, 2010) investigated the effects of adapting the classroom environment on children’s WM skills, measuring effects on all four aspects of WM and far-transfer to vocabulary, reading and maths. Benefits were only reported on the VSSTM measure for one of the cohorts (see Table 3 for details of the study design). The second phase of the study included observation of whether teachers were using the WM strategies. The study authors reported that there was a positive association between teachers’ use of strategies and children’s post-intervention reading and spelling scores but that this association was also seen on pre-intervention measures of reading comprehension, suggesting that the most effective teachers were already using appropriate strategies prior to the intervention. This review has found no evidence that adapting the classroom environment in the absence of direct training improves children’s WM.

2. Direct WM training without strategy instruction

All three studies (Banales et al., 2015, Henry et al., 2014, Passolunghi and Costa, 2016) that implemented a direct WM training approach without strategy instruction reported improvements on trained WM measures. Between study comparisons revealed that all of the intervention ingredients were ELWM tasks and the training produced greater effects on ELWM outcomes than on STM skills. Evidence for near- and far-transfer effects was variable. Banales et al. (2015) did not find any transfer from verbal ELWM training to reading skills. Henry et al. (2014) did find near-transfer effects from trained ELWM tasks to some (but not all) untrained STM skills and to an untrained ELWM task (counting recall), but far transfer effects to single word reading and maths were absent (although there was some evidence for a far transfer effect on reading comprehension). Passolunghi and Costa (2016) reported far transfer effects on numeracy skills following training on all four aspects of WM but they did not measure near-transfer effects. The results here show that non-computerised direct WM training produces positive effects on the trained tasks when these are executive-loaded but the evidence for near- and far-transfer effects is mixed.

3. Direct WM training with strategy instruction

Five studies, all with children between 7 and 9 years of age, implemented direct WM training with strategy instruction. Table 4 gives an overview of the WM aspect(s) trained and the strategies taught in these interventions, showing that two studies trained STM tasks and three targeted ELWM tasks.

**Table 4:** Direct WM training with strategy instruction: tasks and taught strategies.

Study author and year	Trained WM tasks	Taught strategies
Caviola et al. (2009)	VSSTM, specifically, sequential-spatial tasks.	– Coding stimuli and analysing information - looking closely at figures, naming and rehearsing labels when following a path. – Chunking visuospatial stimuli. – Using mental images to execute a task. – Verbalising mental images.
Comblain (1994)	VSTM tasks: Digit span Word span Letter span	Described as rehearsal
Cornoldi et al. (2015)	VELWM: variations of classical WM tests e.g. listening span VELWM – recall plus a secondary task	Meta-cognitive strategies focusing on those related to: (1) understanding the wording of a problem (2) improving the visual-schematic representation of problems
Peng and Fuchs (2015)	VELWM tasks: Counting figures Calculation span Operation span Puzzles	Rehearsal strategy training
Witt (2011)	VSTM: Word list recall forwards VELWM: Backward digit recall VELWM: Updating task VELWM: Counting recall	– Imagine a story – Sub-vocal rehearsal – strategies for preventing distraction

The two studies that targeted STM skills produced limited evidence for positive effects on the trained skills. Caviola, Mammarella, Cornoldi, and Lucangeli (2009) found that both the experimental and the control groups improved on the trained VSSTM measure but there was no significant post-intervention difference between the groups. Comblain (1994) reported improvements on trained VSTM tasks but there was no clear comparison with a control group. Training on ELWM tasks produced more consistent data. The studies by Witt, 2011, Cornoldi et al., 2015 demonstrated significant gains for the experimental groups compared to their control conditions, although in both cases they were passive controls. Peng and Fuchs (2015) paper was the only study to compare the effects of WM training with and without strategy use. These two intervention groups were compared to a no-intervention control group. Neither of their experimental groups made significantly greater gains than the control group on the trained ELWM tasks and there was no difference between the strategy and the no-strategy groups.

Three studies in this category (Caviola et al., 2009, Witt, 2011, Peng and Fuchs, 2015) measured near-transfer effects. Caviola et al. (2009) reported improvements on one VSELWM near-transfer task (backward Corsi block tapping task) but did not demonstrate effects on the trained VSSTM task (see above). Similarly, Peng and Fuchs (2015) measured three untrained WM tasks and saw improvements on one of these (listening recall, VELWM, where the strategy group outperformed the control group) but not on the trained task. One study, Witt (2011), found improvements on both the trained task and a near-transfer effect to an ELWM measure (visual patterns task). With regards to far-transfer, three studies measured a range of academic outcomes and all reported substantial effect sizes: Caviola et al. (2009) on arithmetical problem solving, and Cornoldi et al., 2015, Witt, 2011 on maths (addition accuracy). However, Caviola et al. (2009) had not shown effects on the trained task and Cornoldi et al. (2015) had not measured near-transfer effects.

Evidence regarding whether children actually used the taught cognitive strategies was limited with only two studies measuring strategy use. Cornoldi et al. (2015) directly measured this using a meta-cognitive questionnaire to investigate the effects of training on children’ strategy use and reported that this significantly increased. Peng and Fuchs (2015) used detailed training logs and also reported increases in strategy use following training.

Overall, the results of the five studies reviewed in this category suggest that effects on trained WM tasks were observed when ELWM tasks were trained. There is inadequate evidence regarding near-and far-transfer effects and the impact of strategy-instruction due to a lack of comprehensive outcome measurement.

4. (a) Training skills which may indirectly impact on WM: physical activity

For the nine studies in which skills that may indirectly impact WM were trained, the effects on the trained task, as well as the WM outcomes are crucial in determining the effectiveness of the interventions (Melby-Lervåg and Hulme, 2013, Melby-Lervåg et al., 2016, Redick et al., 2015).

Five studies implemented a physical activity intervention with typically developing children aged 8–10 years. None of these referred to WM models or cognitive processing frameworks in their rationale; rather they referred to neuro-developmental evidence for the underlying involvement of the cerebellum and prefrontal cortex in both motor and cognitive tasks. Examining the results of these studies means looking at the exact nature of the trained tasks (ingredients), and in doing so, an important distinction can be drawn between activities that mostly challenge: (a) physical fitness i.e., they raise the heart rate but require limited attentional resources under executive control because they are already learned skills with a degree of automaticity such as running; and (b) motor planning skills i.e., they require considerable executive control due to the novelty of the task, but have low cardiovascular impact such as dribbling a ball while avoiding obstacles; or (c) both physical fitness and motor planning skills which includes many sports.

All of these interventions appeared to involve novel motor planning tasks. Thus, they have been defined here as executive-loaded interventions, although in most cases the study authors did not define them in this way or specify the motor planning requirements of the intervention tasks. For example, Davis et al. (2007) described their intervention as an aerobic exercise program but on closer inspection of the treatment ingredients, they included the teaching of novel motor skills. This intervention was conducted with overweight children (*n =* 94) and found no significant weight reduction. Van der Niet, Smith, Oosterlaan, and Scherder (2016) did not find any improvements on children’s physical fitness following their intervention, described as a cognitively demanding exercise program and they did not measure the children’s motor skills. Three studies did report significant improvements on the trained physical activities. Kamijo et al. (2011) reported improvements on physical fitness and Alesi, Bianco, Palma, and Pepi (2016) found significant improvements on agility following a football intervention. Koutsandréou, Wegner, Niemann, and Budde (2016) was the only study that directly compared the effects of physical activity with and without an executive skills element. Both groups (cardiovascular and motor exercise) showed specific improvements from the intervention compared to a no-intervention control group.

Looking at the effects of these physical activity interventions on WM, Davis et al. (2007) found no significant WM gains (though they report positive effects on other aspects of cognitive functioning). Van der Niet et al. (2016) reported significant improvements on VELWM (backward digit span task) but not on VSELWM (visual memory span). The study authors suggested improvements in WM skills, in the absence of enhanced physical fitness, were owed to the cognitively engaging aspect of the trained task. Kamijo et al. (2011) reported mixed findings on VELWM outcomes depending on the complexity of the task. Alesi et al. (2016) also found mixed effects on WM measures, with gains observed on a visuospatial STM task, but not on verbal STM or ELWM measures. Koutsandréou et al. (2016) found positive effects on WM following both the cardiovascular and the motor exercise programs, but to a larger degree from the motor exercise intervention.

To summarise, all of the physical activity interventions reviewed included tasks requiring novel motor planning. Three out of the five studies reported improvements on these executive-loaded skills. The WM outcomes measured varied greatly between studies and verbal ELWM skills were measured most frequently. Four out of the five papers reviewed found significant gains on these outcomes.

(b) Training skills which may indirectly impact on WM: phonological awareness

Two studies investigated the effects of training phonological awareness on WM and both used the Baddeley and Hitch (1974) model as their theoretical framework, hypothesising about the role of phonological processing and memory in language learning (see Table 3). Melby-Lervåg and Hulme (2010) compared the effects of phoneme awareness training, rhyme training and vocabulary training on these trained skills and on WM (serial recall and free recall). Phoneme awareness training improved phoneme awareness skills, benefitted serial recall and had a smaller positive effect on free recall. Rhyme training improved rhyme generation skills but had no impact on serial or free recall. Vocabulary training improved children’s ability to define the trained words. It improved free recall and had a smaller positive effect on serial recall, but again only for the trained words. Van Kleeck, Gillam, and Hoffman (2006) reported WM outcomes for children with language impairment (*n =* 24) who had taken part in an intervention targeting phoneme awareness and rhyme skills (see Van Kleeck, Gillam, & McFadden, 1998). The original paper reported that children made significant gains from pre- to post-intervention assessment on rhyme and phoneme awareness tasks, but when comparisons were made with the control group, the gains on rhyme could not be attributed to the intervention. In the earlier paper, the study authors reported significant pre- to post-intervention improvements on VSTM measures but in the more recent paper there was no comparison with a control group. When taken together, the results of these two studies suggest that rhyme and vocabulary training do not improve VSTM skills but phoneme awareness training produced positive effects.

(c) Training skills which may indirectly impact on WM: play and inhibition

Two studies implemented small group interventions with 4–5 year olds. Thibodeau et al. (2016) studied the effects of a fantastical play intervention and Volckaert and Noël (2015) investigated the effects of training exercises that tapped into four aspects of inhibitory control: interruption of an ongoing response; impulsivity control; inhibition of a predominant response; and inhibition of external factors. Both studies reported significant improvements on the trained tasks (fantastical play and inhibition). Thibodeau et al. (2016) found positive effects on VSTM and Volckaert and Noël (2015) reported gains on a factor analysis combining three WM outcomes (VSSTM, VELWM and VSSTM). They also included far-transfer measures of children’s externalising behaviours and reported significant effects including a reduction in children’s negative and inattentive behaviours. The study authors suggested that a meta-cognitive element to the intervention may have supported transfer to attention behaviours. To summarise, these studies suggest that children’s fantastical play skills can be improved by intervention and that this can strengthen VSTM skills. Inhibitory control was improved by training and produced effects on WM, attention and behaviour.

### Synthesized findings; active ingredients, dosage, transfer effects and maintenance

The current section provides a synthesis of the results for: the primary outcomes (effects on trained aspects of WM, research objective 2); the secondary outcomes (near- and far-transfer effects, research objective 3); and durability over time (research objective 4). Table 5 presents the intervention ingredients extracted from those studies deemed to be effective, following the criteria outlined in the data analysis section.^4^ The key finding is clearly demonstrated - in all of the effective interventions, the trained tasks were executive-loaded. Overall, the evidence for near- and far-transfer effects from WM interventions within the child’s environment was limited to three studies (see Table 5: Henry et al., 2014, Volckaert and Noël, 2015, Witt, 2011), although the evidence was constrained by the lack of near-transfer effects being measured in some studies. For example, Peng and Fuchs, 2015, Cornoldi et al., 2015, Passolunghi and Costa, 2016 reported significant far transfer effects following training, but had not measured near-transfer effects.

**Table 5:** Synthesized findings for effective interventions on trained aspects of WM, near- and far-transfer effects.

Outcomes measured	Intervention demonstrating positive effects			Dosage			
				Dose	Dose frequency		Total intervention duration
					Session duration (min)	Session frequency (times per week)	
	Trained task	Executive-loaded?	Study author and year				
Trained aspects of working memory	Direct ELWM training: Odd one out and listening recall	✓	Henry et al. (2014)	22 trials (11 of each task)	10	3	6 weeks
	Direct ELWM training: Verbal and visuospatial dual tasks	✓	Passolunghi and Costa (2016)	?	60	2	5 weeks
	Direct ELWM training: word list updating	✓	Cornoldi et al. (2015)	?	60	1	8 weeks
	Direct ELWM training with strategy instruction: backward digit recall with rehearsal	✓	Witt (2011)	?	15	?	6 weeks
	Phoneme awareness	✓	Melby-Lervåg and Hulme (2010)	?	7	1	10 consecutive school days
		✓	Van Kleeck et al. (2006)	?	15	2	12 weeks
WM impacted indirectly by training other skills	Cognitively-demanding physical activity	✓	Alesi et al. (2016)	?	75	2	6 months
		✓	Kamijo et al. (2011)	?	120	1	150 days (9 months)
		✓	Koutsandréou et al. (2016)	?	45	3	10 weeks
		✓	Van der Niet et al. (2016)	?	45	3	10 weeks
	Fantastical play	✓	Thibodeau et al., 2016	?	15	5	5 weeks
	Inhibition	✓	Volckaert and Noël (2015)	?	15	?	6 weeks
Near-transfer measures^a^	Direct ELWM training: Odd one out and listening recall. Near-transfer to another ELWM task (counting recall)	✓	Henry et al. (2014)	22 trials (11 of each task)	10	3	6 weeks
	Direct ELWM training with strategy instruction: backward digit recall with rehearsal. Near-transfer to VSSTM.	✓	Witt (2011)	?	15	?	6 weeks
Far-transfer measures	Direct ELWM training: Odd one out and listening recall Improvements on one measure of reading comprehension.	✓	Henry et al. (2014)	22 trials (11 of each task)	10	3	6 weeks
	Direct ELWM training with strategy instruction: backward digit recall with rehearsal. Far-transfer to numeracy skills (addition).	✓	Witt (2011)	?	15	?	6 weeks
	Inhibition. Far-transfer - a reduction in inattention levels and negative reactions in games.	✓	Volckaert and Noël (2015)	?	15	?	6 weeks

a Caviola et al. (2009) reported gains on one of the four untrained WM skills they measured (backward Corsi task, VSEWLM) but there were no effects on the trained VSTM skill.

There was less consistency regarding the findings on intervention dosage. When they were reported, there was considerable variation between studies. Dose/number of trials per intervention session was only reported in one paper (Henry et al., 2014). Despite this, Table 5 demonstrates that training effects can be observed following relatively short interventions of 5–8 weeks (albeit with varying intensity of sessions per week) (see Appendix A for full details).

The fourth research objective was to identify whether WM gains were durable over time. Only four of the included studies (Banales et al., 2015, Henry et al., 2014, Cornoldi et al., 2015, Comblain, 1994) included any follow-up period so the evidence here is limited. These studies were heterogeneous in their intervention approaches and in the length of time between post-intervention assessment and follow up but all demonstrated that WM gains had been maintained.

### Risk of bias

The risk of bias in the included studies is significant due to methodological issues (see completed Cochrane risk of bias tool, Appendix B). Fewer than 50% of included studies were randomised controlled trials (*n =* 8) and none of the studies reported undertaking a power analysis, so small sample sizes may have resulted in inflated effects being reported (Button et al., 2013). Whether the children’s teachers were aware of the purpose of the intervention was often unclear and without clear audit protocols being put in place this raises questions about the fidelity of implementation. There was also a lack of active control groups and blinding, which are important in WM research studies, especially with children whose familiarity with experimenters could affect their motivation and outcomes (Melby-Lervåg and Hulme, 2013, Melby-Lervåg et al., 2016, Redick et al., 2015). Reporting issues were also identified by the CONSORT (Schulz et al., 2010) and TREND (Des Jalais et al., 2004) checklists especially in relation to randomisation and allocation processes which were consistently unreported. Publication bias may also threaten the validity of the findings because, although the grey literature was comprehensively searched, only published papers met the review criteria. On balance, the review findings for the effectiveness of non-computerised WM interventions in children’s everyday contexts should be considered as suggestive evidence and viewed with some caution until further evidence is obtained from more robust studies.

## Discussion

The aim of this review was to examine systematically the effectiveness of interventions targeting WM in 4–11 year olds applied within their everyday contexts. The first research objective was to identify both the types of WM interventions implemented and their theoretical underpinnings. A systematic search of the literature resulted in eighteen studies being reviewed, encompassing a range of intervention approaches including: adapting the environment to reduce WM loads; direct WM training without strategy instruction; direct WM training with strategy instruction; and training skills that may indirectly impact on WM (physical activity, phonological awareness, fantastical play and inhibition). Many of the included studies lacked a clear theoretical account of why the intervention should impact WM. In particular, the studies looking at indirect training of WM through physical activity, fantastical play (Thibodeau et al., 2016), or inhibition (Volckaert & Noël, 2015) were not explicitly underpinned by theoretical models of WM.

The second research question was to identify the effects of interventions targeting WM in children’s everyday contexts on each aspect of WM. Answering this question was challenging because in the majority of studies, the aspect of WM being trained or measured was not elucidated, and there was often no clear distinction between STM and ELWM tasks. Nonetheless, a significant outcome of this review is that WM skills can be altered through diverse interventions, particularly in relation to verbal WM skills which were more frequently measured than the visuospatial domain. ELWM skills appear to be more amenable to change than STM skills.

The third and fourth research questions were to identify any near-and far-transfer effects and the durability of WM gains over time. The evidence here was limited because few studies measured these outcomes. However, there is preliminary evidence suggesting that certain direct and indirect WM tasks applied within children’s everyday contexts have the potential to produce: near-transfer effects on similar WM tasks (Henry et al., 2014, Witt, 2011); and far-transfer effects on areas such as reading comprehension (Henry et al., 2014), numeracy skills (Witt, 2011), attention and behaviour (Volckaert & Noël, 2015).

Reflection on the potential of these varied interventions to produce training and transfer effects leads to questions about which children may benefit most and the impact of individual differences. The age of participants varied within each intervention type, making it difficult to draw strong conclusions about age effects. A number of the interventions reviewed showed significant benefits for younger children (4–5 year olds) (Passolunghi and Costa, 2016, Thibodeau et al., 2016, Van Kleeck et al., 2006, Volckaert and Noël, 2015), reinforcing the idea that non-computerised approaches might be more suitable for younger children. The majority of studies were with typically developing children, meaning it was not possible to evaluate the effectiveness of the interventions for children with identified WM difficulties, neurodevelopmental difficulties or at-risk groups such as those from low-socioeconomic backgrounds persist. Nonetheless, it would be beneficial in future reviews to evaluate the effects of differences in baseline abilities and other individual differences.

The final research question concerned the active ingredients and optimum dosage requirements for WM interventions applied in children’s everyday contexts. Here the synthesized data from those studies demonstrating effects on WM indicated that the most effective tasks were executive-loaded i.e., they tap into attentional resources under executive control. The effective ELWM tasks were: listening recall, odd one out, backward digit recall, verbal and visuospatial dual tasks, and word list updating. When considering indirect WM tasks, there is suggestive evidence that cognitively-demanding physical activity (motor planning), fantastical play (Thibodeau et al., 2016) and inhibition training (Volckaert & Noël, 2015) are beneficial to ELWM. In these effective interventions, it is perhaps the overlapping nature of the trained activities, WM and real-world skills that is the active ingredient resulting in promising positive effects (Gathercole et al., 2018).

The optimum dosage required to produce training effects remains uncertain because dosage variables were often unreported or showed significant variation across studies. However, relatively short interventions of 5–6 weeks in total duration (albeit with different frequency of sessions) were shown to be effective (e.g., Henry et al., 2014, Thibodeau et al., 2016, Volckaert and Noël, 2015). There is still a great deal to learn about optimal levels of dosage for interventions in the area of child language and development (Justice, 2018). Clearly, this is an area requiring more detail and rigour in intervention studies to better inform our understanding of dosage and increase applicability of interventions to clinical contexts (Sugden, Baker, Munro, Williams, & Trivette, 2018).

### Limitations

Several methodological factors may limit confidence in the findings of this review, including the risk of bias in individual studies due to the frequent lack of control of confounding variables, small samples sizes and an absence of blinding of participant and outcome assessors. Only two of the included studies incorporated active control groups in their design. This highlights a significant weakness that has also impacted on empirical research into computerised WM training (Melby-Lervåg & Hulme, 2013). The lack of an active control group means that training effects could be attributed to other variables that differ between experimental and passive control groups (Redick et al., 2015).

The absence of a clear theoretical framework in many of the included studies has limited the evidence found in this review. The lack of a sound theoretical underpinning leads to ambiguous predictions and unclear conclusions about training effects, which then impact on outcome measurement. The distinction between STM and ELWM tasks is widely accepted across both componential and attentional models of WM (Baddeley and Hitch, 1974, Baddeley, 2000, Cowan, 2005). However, in many studies the trained tasks and outcomes measured were not clearly defined. For example, none of the physical activity studies measured VSELWM which, given Baddeley, 2000, Baddeley, 2007 hypothesis that kinaesthetic memory is managed within the visuospatial sketchpad, may also be expected to show associations with motor planning. Unspecified causal pathways also resulted in near-transfer effects being measured inadvertently or, in many cases, not measured at all. The lack of recognition of near-transfer effects subsequently impacts on conclusions about far-transfer. It is difficult to attribute far-transfer effects to interventions if near-transfer effects have not been demonstrated (Redick et al., 2015). Only one study explicitly recognised this (Henry et al., 2014), with three others measuring far-transfer effects without measuring near-transfer (Banales et al., 2015, Cornoldi et al., 2015, Passolunghi and Costa, 2016).

The clinical diversity of the included interventions is both a limitation and a strength of this review. The heterogeneity of the interventions implemented and the study design (randomised and non-randomised) means that a meta-analysis was not appropriate in this review. This may have limited the strength of the evidence presented but synthesizing study effects in a meta-analysis would have produced misleading findings. The diverse nature of the interventions reviewed has enabled the identification of key ingredients, notably training ELWM, as well as developing recommendations that can help inform novel methodologies (Reeves et al., 2008) and significantly enhance thinking to support the third wave of WM research (Morra & Borella, 2015).

### Future directions

From both theoretical and applied perspectives, many questions about the utility of WM training approaches remain unanswered. We must therefore consider whether it is overly optimistic to continue developing and testing diverse, WM intervention approaches with the aim of impacting on real world skills, and indeed whether this is a worthy goal for applied research. This review provides suggestive evidence for the effectiveness of diverse WM interventions applied within children’s everyday context, when the trained tasks are executive-loaded. Evidence has emerged of what might be possible and there are several advantages to the types of interventions reviewed here in comparison to computerised WM training. These include: greater flexibility in how the tasks are presented; less of a requirement for young children to sit still for long periods of time; and opportunities to promote social and emotional development in activities embedded within the child’s own environment (Diamond & Lee, 2011) (which may be appealing to education practitioners). It has also been speculated that young children may not be motivated by this approach (Jaeggi et al., 2012, Wass et al., 2012).

The considerable challenges of conducting further research in applied settings must also be acknowledged. Although computerised WM training lacks the ecological validity of WM interventions provided within the child’s everyday context, it allows for tighter control over both the training environment and paradigms. The manipulation of WM loads on a trial-by-trial basis may be important for improving WM (Klingberg, 2010), and this is easier with computerised programmes. Ensuring that children are working at capacity is much harder to achieve in real-life contexts, particularly when working with groups. Conversely, there is some evidence to suggest that the incremental increases automatically prescribed during computerised training can be too great, thereby lessening children’s motivation (Jaeggi, Buschkuehl, Jonides, & Shah, 2011). Controlling the level of distraction in the environment is also difficult in non-laboratory training environments. However, the occurrence of distraction alongside training may indeed enhance its ecological validity and support transfer effects (De Simoni & von Bastian, 2018). Of course, it may eventually be possible to develop a hybrid intervention approach that integrates the benefits of computerised training in everyday contexts. There may be potential for the utilisation of technology to monitor children’s responses and continually adapt the difficulty level of training trials delivered face-to-face. For example, education professionals could deliver practice trials on ELWM tasks in schools and record responses on a portable device (laptop or tablet) with software informing the facilitator about levels of difficulty.

The strength of evidence found in this review is compromised by inter-related theoretical and methodological issues, a lack of clear empirical evidence does not necessarily equate to ineffectiveness and says nothing about the potential for future interventions (Shipstead et al., 2012). In future research, there needs to be greater recognition that all interventions are based on causal assumptions (Moore & Evans, 2017). Future studies need to present a clear theoretical account of how and why the intervention should impact WM in hypothesis-driven research. It will be important also to apply greater rigour in study methodology, including the use of active control groups and randomised controlled designs. In order to develop a greater understanding of the relationship between intervention ingredients, WM outcomes and real world skills, outcome measurement must be comprehensive. Greater attention must also be paid to intervention dosage. There is a need to look more closely within interventions, to distil them and identify exactly which ingredients and dosages act as optimal mechanisms of change. This will require replicating specific tasks or subgroups of tasks embedded in successful interventions and varying the doses delivered in treatment sessions, frequency of intervention sessions, and overall duration of intervention in comparative studies (Warren et al., 2007).

Clarifying for whom non-computerized WM interventions may be most effective, and in what circumstances, should be a goal of further research. Future studies should target children from understudied sub-groups such as those with identified WM difficulties, neurodevelopmental difficulties or at-risk groups such as those from low-socioeconomic backgrounds. Greater consideration should be given to the role of individual differences that could influence the effectiveness of interventions e.g., cognitive level, motivation (Melby-Lervåg et al., 2016). It may also be pertinent to consider the context in which the intervention is implemented such as the influence of the child’s environment (Hawe, 2015, McLeroy et al., 1988).

## Conclusions

Diverse interventions applied within young children’s everyday contexts, have produced improvements on their WM skills and have the potential to produce near- and far-transfer effects. Both direct training on WM tasks and practicing certain skills that may indirectly impact on WM (physical activity, fantastical play and inhibition) were beneficial. The common ingredient across effective interventions was the executive-loaded nature of the trained task i.e., training on a task that taps into attentional and processing resources under executive control not just the storage of information. The strength of the evidence is tempered by a lack of clear theoretical underpinnings, rigorous methodology and consideration of dosage. Further well-designed and controlled studies with sound theoretical underpinnings and comprehensive outcome measurement, comparing carefully-considered dosages, are required to expand and enhance the evidence base.

## Funding

This systematic review was undertaken as part of a doctoral research study funded by the Research and Development Division of the Public Health Agency, Northern Ireland. The funder has not been involved at any stage of the review process. The authors alone have been responsible for the design, conduct of the review, analysis and interpretation of the findings, writing of the report and the decision to submit this article for publication. Open access was paid for by the Medical Research Council of the United Kingdom, grant code RG91365.

## Declaration of interest

None.

## Figures and Tables

**Table:** 

Non-computerised WM intervention approach	Paper	Rationale provided for dosage	Dosage				Do study authors reflect on dosage in their discussion?
			Dose	Dose frequency		Total intervention duration	
				Session duration (mins)	Session frequency (times per week)		
Adapting environment	Elliott et al. (2010)	No	No details provided to quantify amount of teacher training provided. Specifying dosage for the children in this study is not applicable due to the nature of the intervention			8–11 months	No
Direct WM without strategy training	Banales et al. (2015)	No	?	30	3	8 weeks	They question if the amount of training was sufficient but do not comment on the impact of the intervention being delivered via Skype
	Henry et al. (2014)	Yes. Number of sessions chosen to conform to computerised training studies	22 trials (11 of each task)	10	3	6 weeks	Yes. They suggest the brevity of each session supported children’s compliance, enjoyment and motivation and contributed to effectiveness of the intervention
	Passolunghi and Costa (2016)		?	60	2	5 weeks	No
Direct WM training with strategy training	Caviola et al. (2009)	No	?	40 (plus 10 min discussion)	2	4 weeks (7 sessions)	No
	Comblain (1994)	Yes. Inspired by methodology in a previous study	?	30	1	8 weeks	No
	Cornoldi et al. (2015)	No	?	60	1	8 weeks	No
	Peng and Fuchs (2015)	No	?	35	1 per day	10 consecutive days	Yes. They question if the amount of training was sufficient
	Witt (2011)	No	?	15	?	6 weeks	No
Training skills which may indirectly impact on WM: phonological awareness	Melby-Lervåg and Hulme (2010)	No	?	7	1	10 consecutive days (Mon- Fri for 2 school weeks)	No
	Van Kleeck et al. (2006) Dosage data extracted from intervention study (Van Kleeck et al. (1998)	No.	?	15	2	12 weeks each of rhyme & phoneme awareness training (24 weeks total)	The authors suggest the brevity of the brevity of the intervention sessions make it attractive and replicable. No discussion on the effectiveness of the dosage provided
Training skills that may indirectly impact on WM: physical activity	Alesi et al. (2016)	No	?	75	2	6 months	No
	Davis et al. (2007)	Yes. Inspired by methodology in a previous study	?	Low dose – 20 High dose – 40	5	15	Yes. They suggest that the total intervention duration may have been too short and reported that the high dose condition was more effective
	Kamijo et al. (2011)	No	?	120	1	150 days (9 months)	No
	Koutsandréou et al. (2016)	No	?	45	3	10 weeks	No
	Van der Niet et al. (2016)	No	?	30	2	22 weeks	Yes. Authors suggest that lack of effect on physical fitness may be related to the frequency or intensity of the intervention
Training skills that may indirectly impact on WM: play	Thibodeau et al. (2016)	Yes. Number of sessions chosen to conform to computerised training studies	?	15	5	5 weeks	No
Training skills that may indirectly impact on WM: inhibition	Volckaert and Noël (2015)		?	45	2	8 weeks	Yes. The authors reported moderate effect sizes and suggest that more than 16 sessions may be required to obtain larger effects

*Note:* Cumulative intervention intensity (dose × dose frequency × total intervention duration) was omitted from this table as it was only calculable for one study (Henry et al., 2014) which provided the number of trials per session (dose). Cumulative intensity for Henry et al. (2014) = 396 (22 × 3 × 6).

**Table:** 

Study author and year	Random sequence generation (RCTs)/Allocation	Allocation concealment	Blinding of participants an personnel	Blinding of outcome assessment	Incomplete outcome data – trained WM	Selective reporting
Elliott et al. (2010)	High	Unclear	High	Unclear	Unclear	Unclear
Banales et al. (2015)	High	Unclear	Low	High	Low	Unclear
Henry et al. (2014)	Unclear	Unclear	Low	Low	Low	Low
Passolunghi and Costa (2016)	Unclear	Unclear	Low	Low	Low	Unclear
Caviola et al. (2009)	High	Unclear	Low	Low	Unclear	High
Comblain (1994)	High	Unclear	Unclear	High	Unclear	Unclear
Cornoldi et al. (2015)	High	Unclear	High	Unclear	Low	Unclear
Peng and Fuchs (2015)	Unclear	Unclear	Unclear	Low	High	Unclear
Witt (2011)	High	Unclear	High	Unclear	Unclear	Unclear
Melby-Lervåg and Hulme (2010)	High	Unclear	Unclear	Unclear	High	Unclear
Van Kleeck et al. (2006)	High	Unclear	Unclear	Unclear	High	High
Alesi et al. (2016)	High	Unclear	Unclear	Unclear	Low	Unclear
Davis et al. (2007)	Unclear	Unclear	Unclear	Low	Low	Unclear
Kamijo et al. (2011)	Unclear	Unclear	Unclear	Unclear	High	Unclear
Koutsandréou et al. (2016)	Unclear	Unclear	Unclear	Low	Unclear	Unclear
Van der Niet et al. (2016)	High	Unclear	Unclear	Low	Low	Unclear
Thibodeau et al. (2016)	Unclear	Unclear	Low	Low	Unclear	Unclear
Volckaert and Noël (2015)	High	Unclear	Unclear	Low	N/A	Unclear

## Footnotes

1 **Abbreviations:** WM: working memory; ELWM: executive-loaded working memory; STM: short-term memory; VSTM verbal short-term memory; VSSTM: visuospatial short-term memory; VELWM verbal executive-loaded working memory; VSELWM visuospatial executive-loaded working memory.

2 Following international consensus, specific language impairment is now referred to as developmental language disorder (Bishop, Snowling, Thompson, Greenhalgh, & The CATALISE Consortium, 2016).

4 Comprehensive summaries of the training effects for these outcomes can be found in the supplementary materials (Tables B1–B5)**.**

## Footnotes

3 Due to the overlapping nature of executive functions, it could be argued that training inhibitory control should be considered as direct WM training. This study (Warren et al., 2015) was categorised as indirectly targeting WM as the intervention did not involve repeated practice on memory tasks.
