# Supplementary material for: Interventions targeting working memory in 4–11 year olds within their everyday contexts: A systematic review
Source: Dev Rev. 2019 Jun;52:1–23. doi: 10.1016/j.dr.2019.02.001 (PMC6686208; doi:10.1016/j.dr.2019.02.001)
Supplement: Supplementary data 2 [file mmc2.docx]

**Supplementary Material: Table A. Outcomes measured and results of each included study (*n=* 18)**

| **Study author, year and intervention approach** | **PRIMARY OUTCOME** | | | | **SECONDARY OUTCOMES** | | | **Main findings** |
| --- | --- | --- | --- | --- | --- | --- | --- | --- |
|  | **WM outcomes measured** | | | | **Near-transfer**  **effects**  **(untrained WM)** | **Far- transfer effects** | **Durability**  **(follow-up period)** |  |
|  | **Verbal STM** | **Verbal**  **ELWM** | **Visuo**  **Spatial**  **STM** | **Visuo**  **Spatial**  **ELWM** |  |  |  |  |
| **Elliott et al.**  **(2010)**  **Adapting the environment** | Digit recall  Word recall  Non-word recall | Backward digit recall  Listening recall  counting recall | Dot matrix  Mazes memory  Block recall | Odd one out  Mr X  Spatial recall | _ | 5- 6yr olds– vocabulary  9- 10 yr. olds –reading and maths | _ | **WM measures:** no significant gains on any WM measures except for dot matrix (VSSTM) with one cohort of the study.  **Far-transfer:** no gains found. |
| **Banales et al. *(*2015)**  **Direct WM training without strategy instruction** | _ | **Verbal N-back**  **Listening recall** | _ | _ | _ | Reading: sight word recognition and word decoding | 8 weeks | **Trained ELWM tasks:** Two children demonstrated improvement on listening recall but not on N-back task.  **Far-transfer**: no effect of WM training.  **Durability** outcomes measured 8 weeks’ post-intervention. One child who had improved on the listening recall task had maintained progress. None showed any delayed gains on reading skills. |
| **Henry et al.**  **(2014)**  **Direct WM training without strategy instruction** | Digit recall   - Word recall | **Listening recall**  Counting recall | Block recall | **Odd one out** | Digit recall (VSTM)  Word recall (VSTM)  Block recall (VSSTM)  Counting recall (VELWM) | Number skills  Spelling  Reading comprehension | 6 months post-intervention: all WM measures  (trained tasks and near-transfer), word reading and number skills assessments.  12 months post-intervention: two further far-transfer measures included (spelling, reading comprehension). | **Trained VELWM and VSELWM tasks:** significant post-training differences between intervention and control groups in favour of experimental group. Baseline to post-intervention change was greater for experimental group.  **Near-transfer:** mixed results on untrained STM skills- intervention group showed no advantage on block recall (VSSTM) or digit recall (VSTM) but on word recall (VSTM) they performed significantly better than the control group. Untrained VELWM measure (counting recall) - trained group out-performed control group.  **Far-transfer**: no significant effects of the intervention were found.  **Durability**: 6-month follow-up- gains on trained WM and near-transfer measures were maintained; 12 month follow-up - intervention group scored significantly higher than control group on reading comprehension. |
| **Passolunghi and Costa (2016)**  **Direct WM training without strategy instruction** | - **Word recall** | **Verbal dual task** | **Pathway recall (spatial)** | **Visuospatial dual task** | _ | Numeracy | _ | **Trained tasks**: no significant effect on verbal or visuospatial STM measures. On ELWM tasks - WM training group made greater gains than the control and numeracy groups.  **Far-transfer:** WM training group and numeracy group made greater gains than the control group. No difference between the two intervention groups. |
| **Caviola et al*.* (2009)**  **Direct WM training with**  **strategy instruction** | Forward digit span | Backward digit | Visual patterns test (visual)  **Corsi block tapping (spatial)** | Backward Corsi | Forward digit (VSTM)  Backward digit (VELWM)  Visual patterns test (VSSTM)  Backward Corsi (VSELWM) | _ | _ | **Trained VSSTM task**: experimental and control groups improved. No post-intervention difference between groups.  **Near-transfer:** post intervention gains found on backward Corsi task. No benefit of training found on other near-transfer measures.  No assessment of children’s strategy-use. |
| **Comblain (1994)**  **Direct WM training with**  **strategy instruction** | **Digit span,**  **Letter span and**   - **Word span** | _ | _ | _ | _ | ­_ | 6 weeks  6 months | **Trained VSTM tasks:** significant improvements on three immediate post-intervention measures. No clear comparison with control group reported.  **Durability:** performance decreased between 6 week and 6-month assessment points but remained significantly higher than pre-intervention level. |
| **Cornoldi et al. (2015)**  **Direct WM training with**  **strategy instruction** | _ | **Word list recall tasks with updating** | _ | _ | _ | Arithmetical problem solving skills | Training group 1 - 3 months post training).  No follow up for training group 2 due to cross-over study design. | **Trained ELWM tasks:** both groups improved from baseline scores when tested immediately after intervention period. No gains observed during untrained periods.  **Far-transfer**: both groups improved during trained periods and not during untrained periods. Regression analyses indicated the only significant predictor of problem solving was WM updating.  **Durability:** training group 1 maintained gains 8 weeks later. No further improvement in WM skills.  Meta-cognitive questionnaire used to investigate effects of training on strategy use: significant increases during training periods which were maintained by group 1. |
| **Peng and Fuchs (2015)**  **Direct WM training with**  **strategy instruction** | Digit recall | Listening recall  **Counting recall (named counting figures in the intervention description)** | Block recall | _ | Digit recall (VSTM)  Listening recall (VELWM)  Block recall (VSSTM) | Listening comprehension and retell.  Assessed  using 2 scores from the Qualitative Reading Inventory (QRI) (Leslie and Caldwell, 2001) | _ | **Trained task** all three groups (strategy instruction; no strategy instruction and no intervention) improved from pre- to post-intervention assessment. When compared to no-intervention control group - neither strategy instruction nor no-strategy instruction groups made significant gains. No difference between the strategy and no-strategy groups.  **Near-transfer:** No significant group differences post-intervention except on listening recall task where strategy group outperformed the control group.  **Far-transfer:** QRI Retell measure- strategy group significantly outperformed the control group. No-strategy group did not  QRI listening comprehension measure, both intervention groups out-performed control condition.  Strategy use: children in the strategy instruction group were observed using rehearsal on 89% of trials compared to 17% for the no-strategy group. |
| **Witt (2011)**  **Direct WM training with**  **strategy instruction** | _ | **Backward digit recall** | Visual patterns test | _ | Visual patterns (VSSTM) | Maths: addition accuracy and time | _ | **Trained VELWM task:** intervention group showed greater improvements on post-intervention assessment than the matched-pair control group.  **Near-transfer:** intervention group showed greater improvements on post-intervention assessment than the control group.  **Far-transfer:**  intervention group showed greater improvements on post-intervention assessment than the control group on  addition accuracy but there was no change on children’s addition time.  No objective measurement of children’s strategy use but the study author reported that some children used the strategies whereas others appeared to be resistant to them. |
| **Alesi et al. (2016)**  **Training skills that may indirectly impact on WM:**  **physical activity** | Digit span | Backward digit | Corsi block tapping | _ | _ | _ | ­_ | **Trained tasks (motor skills**): pre-post intervention assessments on an agility test (Alesi *et al.,* 2014) indicated significant improvements for the football group whereas there was no effect for the control group.  **WM measures:** Mixed results Significant pre- to post- intervention improvements for the football group on the visuospatial STM task but no effect on the verbal STM and ELWM tasks. The control group did not improve on any WM measures |
| **Davis et al. (2007)**  **Training skills that may indirectly impact on WM:**  **physical activity** | _ | Successive scale of the Cognitive Assessment System (CAS)  (Naglieri and Das, 1997) | _ | _ | _ | _ | ­_ | **Trained tasks (effects on weight and fitness):** Body Mass Index score (BMI) - no group differences post-intervention. Treadmill performance- both intervention groups improved compared to control group.  No difference between the intervention groups.  **WM measures:** No significant group differences on post-intervention assessment. |
| **Kamijo et al. (2011)**  **Training skills that may indirectly impact on WM:**  **physical activity** | _ | Modified Sternberg  (Sternberg, 1966).  Children had to encode a memory set of one, three or five letters | _ | _ | _ | _ | ­_ | **Trained task (fitness):** Fitness measured by oxygen consumption during treadmill task- Significant pre-post intervention effect for the trained group. No effect for the control group.  **WM measures**: results dependent on task demands. One letter condition - no differences in pre-to post-intervention for intervention or control group. Three letter condition –intervention group’s response accuracy improved. Control group did not. Five letter condition - no improvement in accuracy for either group. |
| **Koutsandréou et al. (2016)**  **Training skills that may indirectly impact on WM: physical activity** | _ | Letter digit span** | _ | _ | _ | _ | ­_ | **Trained tasks (physical fitness and motor performance):** fitness level of  cardiovascular exercise (CE) group significantly improved. Motor demanding exercise (ME) and control groups did not. Motor performance- all 3 groups improved. ME group significantly better than control group on post-test. CE group were not.  **WM measures:** CE and ME groups improved. Control group did not. Between group comparisons indicated ME group significantly better than controls but not significantly better than the CE group. Supplementary analysis indicated pre-post intervention difference was significantly larger in ME group compared to CE group. CE improved more than controls. WM benefited from both cardiovascular and motor exercise programs, but to a larger degree from the motor exercise intervention. |
| **Van der Niet et al. (2016)**  **Training skills that may indirectly impact on WM:**  **physical activity** | _ | Backward digit | _ | Visual memory span | _ | _ | ­_ | **Trained task (physical fitness):** No effects found on any physical fitness variables. Motor skills not assessed.  **WM measures:** the intervention group achieved significantly higher post-intervention scores than the no intervention control group on post-intervention assessment of backward digit span (VELWM) but not on visual memory span (VSELWM). |
| **Melby-Lervåg and Hulme (2010)**  **Training skills that may indirectly impact on WM:**  **phonological awareness** | Word span | _ | _ | _ | _ | _ | ­_ | **Trained tasks:** phoneme and rhyme groups improved on trained and untrained words. Vocab group improved on trained words only.  **WM measures:** VSTM measures -results differed between serial recall and free recall tasks and between trained and untrained words.  Serial recall with trained words - all three intervention groups improved. Phoneme group significantly outperformed the other conditions. Vocabulary group improved more than control group but rhyme group did not. Free recall with trained words - vocabulary group showed greater gains than all other groups. Phoneme group outperformed the control group, the rhyme group did not. Serial and free recall with untrained words - no significant differences between groups. Results suggest phoneme awareness training impacted on trained skill, had a transfer effect to serial recall and a smaller effect on free recall. Rhyme training improved rhyme generation skills but had no impact on VSTM. Vocabulary training improved free recall and had a smaller effect on serial recall, but only on trained words. |
| **Van Kleeck et al. (2006) (relating to Van Kleeck et al. 1998)**  **Training skills that may indirectly impact on WM:**  **phonological awareness** | Word span  Non-word span | _ | _ | _ | _ | _ | ­_ | **Trained tasks (van Kleeck *et al.,* 1998**):  Intervention group made significantly improved on rhyme and phoneme awareness skills. Gains on rhyme not attributed to intervention as control group also improved.  **WM measures (van Kleeck *et al.,* 2006):** Report significant pre- to post-intervention improvements for the trained children. No comparison with control group |
| **Thibodeau et al. (2016)**  **Training skills that may indirectly impact on WM:**  **fantastical play** | Digit span | _ | _ | _ | _ | _ | ­_ | **Trained task (fantastical play):** fantastical play group engaged in more pretending behaviours than the non-imaginative play and control groups. No differences observed on 4 other constructs of fantasy orientation. Baseline to post-intervention differences observed in fantastical play group. No change in the other 2 groups.  **WM measures:** Fantastical play group improved from pre- to post-intervention. The other 2 groups did not. |
| **Volckaert and Noël (2015)**  **Training skills that may indirectly impact on WM:**  **inhibition** | Word span | Categospan | Corsi block tapping | _ | _ | Measures of children’s externalising behaviours: Unfair Card Game (UCG) (Roskam *et al.,*  2016); Conners Rating Scale (Goyette *et al.,*  1978). | ­_ | **Trained task (inhibition):** experimental group significantly improved from pre- to post-intervention. Control group did not.  **WM measures**: 3 tasks combined into factorial analysis. Experimental group improved from pre- to post- intervention. Control group did not.  **Far- transfer:** UCG - experimental group showed significantly less negative behaviours after training. Control group showed more. Conners scale - parents reported no effects on conduct or impulsivity scale but significant effects on hyperactivity. Teachers (blinded to the children’s group) did not report improvements on conduct or hyperactivity scales. Significant improvement on inattention scale for the experimental group but not for the control group. |

*Key: WM measures highlighted in bold font = trained tasks.*

*** task presented verbally but written response required*
