# Supplementary material for: Interventions targeting working memory in 4–11 year olds within their everyday contexts: A systematic review
Source: Dev Rev. 2019 Jun;52:1–23. doi: 10.1016/j.dr.2019.02.001 (PMC6686208; doi:10.1016/j.dr.2019.02.001)
Supplement: Supplementary data 3 [file mmc3.docx]

**Supplementary Material: Table B1: Synthesized results: verbal STM.**

| **Intervention Type** | **Study author and year** | **Population** | **Intervention** | | **Comparison** | | **OUTCOMES** | | |
| --- | --- | --- | --- | --- | --- | --- | --- | --- | --- |
|  |  | **status**  **age** | **Trained**  **Skill** | **executive-loaded?** | **Study design**  **and comparison** | **No of participants** | **Effect on trained skill** | **VSTM outcome(s) measured** | **Reported effect on VSTM** |
| **Adapting the environment** | **Elliott et al. (2010)** | TD  5- 6 yr. olds;  9- 10 yr. olds. | None | N/A | QE  Behavioural teaching;  No intervention | 256 | No evidence of enhance strategy-use by teachers. | Digit span  Word span  Non-word span | No significant pre-post intervention differences or group differences observed (*p>*.05). |
| **Direct WM training with strategy instruction** | **Caviola et al. (2009)** | TD  8-9 yr. olds | Corsi block tapping | 🗶 | QE  General cognitive strategies | 46 | No | Digit span🞟 | No significant difference found on post-intervention group comparison (*p=*.62). |
|  | **Comblain (1994)** | DS  8 yr. olds | Digit, letter and word span | 🗶 | QE  No intervention | 8* | Directly trained VSTM. | Digit span,  Letter span  Word span | Significant improvement for intervention group (*p<*.05). No clear comparison made with control group. |
|  | **Peng and Fuchs (2015)** | LD  7 yr. olds | 4 tasks described as verbal | ✓ | RCT  Without strategy  No intervention | 58 | No | Digit span | Neither the strategy instruction group (*p=* .29) nor the no-strategy instruction group (*p=*.46) made significant gains when compared to the control group. |
| **Direct WM training**  **without**  **strategy instruction** | **Henry et al.**  **(2014)** | TD  7 yr. olds | Listening recall  Odd one out | ✓ | RCT  Active control | 36 | Yes | Digit span🞟  word span🞟 | Significant post-intervention group differences on word span (*p<*.01). On digit span both groups improved to the same extent. |
|  | **Passolunghi and Costa (2016)** | TD  5yr olds | All 4 aspects of WM | ✓ | RCT  Numeracy training  No intervention | 48 | Significant effects on EWLWM but not on STM tasks. | Word span | No significant difference between groups (*p=*.51) |
| **Training skills which may indirectly impact on WM: physical activity** | **Alesi et al. (2016)** | TD  8-9 yr. olds | Football | ✓ | RCT  Sedentary children | 44 | Yes. Football group improved on agility test (Alesi *et al.,* 2014) (p< .05). Control group did not. | Digit span | No significant post-intervention difference between the groups (p>.05). |
| **Training skills which may indirectly impact on WM: phonological awareness** | **Melby-Lervåg and Hulme (2010)** | TD  4 yr. olds | Rhyme and phoneme awareness and vocabulary | 🗶 | QE  3 exp. groups and one untrained control | 160 | Significant effects on  on trained words | Word span (Serial recall  Free recall) | Significant effects on trained words. Phoneme awareness group significantly better than other conditions. Phoneme group significantly outperformed the other conditions.  Free recall with the trained words - the vocabulary group showed greater gains than all other groups. The phoneme group also outperformed the control group but the rhyme group did not. No significant effects on untrained words. |
|  | **Van Kleeck et al. (2006)** | SLI  4 yr. olds | Rhyme and phoneme awareness | 🗶 | QE  No control for WM outcomes | 24 | Gains in phoneme awareness. Gains in rhyme skills were not attributed to the intervention. | Word span  Non-word span | Significant improvement for intervention group (*p<*.001)** |
| **Training skills which may indirectly impact on WM: fantastical play** | **Thibodeau et al. (2016)** | TD  4 yr. olds | Fantastical play | ✓ | RCT  Non-imaginative play  No-intervention control | 110 | Yes. On one measure of fantasy orientation Not significant on 3 other measures. | Digit span | Significant improvements for fantastical play group: (*p=*.003). No-significant gains for: non-imaginative play group (*p=*.801) or control group (*p=*.522) |
| **Training skills which may indirectly impact on WM: inhibition** | **Volckaert and Noël (2015)** | TD | Inhibition | ✓ | RCT  Handicraft lessons | 47 | Yes | Word span | *WM measure: significant pre-post intervention gains for exp. group (*p<*.001). No significant gains for control group – (*p=*.215). |

*Key: TD = typically developing; DS= Down’s syndrome; SLI = specific language impairment; O = overweight; LD = at risk of learning difficulties; QE= Quasi-experimental; RCT = Randomised Controlled Trial.*

🞟 these were untrained WM measures so are near-transfer

* This study also included older participants. Only the child participants have been considered in this review. ** gains on rhyme were not attributed to the intervention (van Kleeck *et al.,* 1998) *** Results across WM measures were combined into a factor analysis so specific effects on VSTM cannot be distinguished.

**Supplementary Material: Table B2: Synthesized results: visuospatial STM.**

| **Intervention Type** | **Study author and year** | **Population** | **Intervention** | | **Comparison** | | **OUTCOMES** | | |
| --- | --- | --- | --- | --- | --- | --- | --- | --- | --- |
|  |  | **status**  **age** | **Trained**  **Skill** | **executive-loaded?** | **Study design**  **and comparison** | **No of participants** | **Effect on trained skill** | **VSSTM outcome(s) measured** | **Effect** |
| **Adapting the**  **environment** | **Elliott et al. (2010)** | TD  5- 6 yr. olds;  9- 10 yr. olds. | None | N/A | QE  Behavioural teaching;  No intervention | 256 | No evidence of enhanced strategy-use by teachers | Dot matrix | In 1 of the 2 cohorts: WM group significantly improved (p=.004). Other groups did not. |
| **Direct WM training**  **without**  **strategy instruction** | **Henry et al.**  **(2014)** | TD  7 yr. olds | Listening recall  Odd one out | ✓ | RCT  Active control | 36 | Yes | Block recall | Post-intervention group comparisons: not significant. |
|  | **Passolunghi and Costa (2016)** | TD  5yr olds | All 4 aspects of WM | ✓ | RCT  Numeracy training  No intervention | 48 | Improved ELWM skills but not STM | Pathway recall | No significant difference between groups (*p=*.25). |
| **Direct WM training with strategy instruction** | **Caviola et al. (2009)** | TD  8-9 yr. olds | Corsi block tapping | 🗶 | QE  General cognitive strategies | 46 | No. | **Corsi block tapping**  Visual patterns test (VPT) | Corsi block tapping: no significant difference post-intervention group difference (p= .13).  VPT: significant difference (*p=*.27). |
|  | **Peng and Fuchs (2015)** | LD  7 yr. olds | 4 tasks described as verbal | ✓ | RCT  Without strategy  No intervention | 58 | No | Block recall | Neither the strategy instruction group (*p=* .99) nor the no-strategy instruction group (*p=*.48) made significant gains when compared to the control group. |
|  | **Witt (2011)** | TD  9 yr. olds | BDS; updating task; counting recall | ✓ | QE  No intervention | 38 | Yes | Visual patterns test | Significant post-intervention group differences (p<.05) |
| **Training skills which may indirectly impact on WM: physical activity** | **Alesi et al. (2016)** | TD  8-9 yr. olds | Football | ✓ | RCT  Sedentary children | 44 | Yes | Corsi block tapping | Pre-post intervention gains for experimental group (*p<*.05) Not significant for control group |
| **Training skills which may indirectly impact on WM: inhibition** | **Volckaert and Noël (2015)** | TD | Inhibition | ✓ | RCT  Handicraft lessons | 47 | Yes | Corsi block tapping | *WM measure: significant pre-post intervention gains for exp. group (*p<*.001). No gains for control group – (*p=*.215). |

**Supplementary Material: Table B3: Synthesized Findings: verbal ELWM**

| **Intervention Type** | **Study author and year** | **Population** | **Intervention** | | **Comparison** | | **OUTCOMES** | | |
| --- | --- | --- | --- | --- | --- | --- | --- | --- | --- |
|  |  | **status**  **age** | **Trained**  **skill** | **executive-loaded?** | **Study design**  **and comparison** | **No of participants** | **Effect on trained skill** | **VELWM**  **outcome(s) measured** | **Effect** |
| **Adapting the**  **environment** | **Elliott et al. (2010)** | TD  5- 6 yr. olds;  9- 10 yr. olds. | None | N/A | QE  Behavioural teaching;  No intervention | 256 | No evidence of enhance strategy-use by teachers. | Backward digit span, listening recall. counting recall | No significant pre-post intervention differences or group differences observed (*p>*.05). |
| **Direct WM training**  **without**  **strategy instruction** | **Banales et al. (2015)** | R+WM  9 yr. olds | N-back  Listening recall | ✓ | Case series  Each child own matched control | 4 | Directly trained VEWLM. | N-back  Listening recall | 2 children improved on listening recall.  None on N-back. |
|  | **Henry et al.**  **(2014)** | TD  7 yr. olds | Listening recall  Odd one out | ✓ | RCT  Active control | 36 | Directly trained VEWLM. | Listening recall  Counting recall | Significant difference between exp. and control (*p<*.001)  Counting recall (*p<*.05) |
|  | **Passolunghi and Costa (2016)** | TD  5yr olds | All 4 aspects of WM | ✓ | RCT  Numeracy training  No intervention | 48 | Significant effects on EWLWM but not on STM tasks. | Verbal dual task | Significant post-intervention group difference between exp. versus numeracy (*p=*.009); and exp. versus control (*p=*.002) |
| **Direct WM training with strategy instruction** | **Caviola et al. (2009)** | TD  8-9 yr. olds | Corsi block tapping | 🗶 | QE  General cognitive strategies | 46 | No | Backward digit span | Post-intervention group comparisons - not significant (*p=*.10) |
|  | **Cornoldi et al. (2015)** | TD  9 yr. olds | Word list updating | ✓ | QE  Cross over design-  Usual maths lessons | 135 | Directly trained VEWLM. | Word list updating | Pre-post intervention comparisons. Both trained groups significant gains (*p=*.002; *p<*.001) |
|  | **Peng and Fuchs (2015)** | LD  7 yr. olds | Counting recall | ✓ | RCT  Without strategy  No intervention | 58 | No significant group differences | Counting recall  Listening recall | All 3 groups showed significant post-intervention improvement. Counting recall - neither the strategy instruction group (*p=* .45) nor the no-strategy instruction group (*p=*.17) made significant gains when compared to the control group. No difference between the strategy and the no-strategy groups (*p=*.52). |
|  | **Witt (2011)** | TD  9 yr. olds | Backward digit span, Updating and counting recall | ✓ | QE  No intervention | 38 | Significant post-intervention group difference (*p<*.05) | Backward digit span | Significant post-intervention group difference (*p<*.05) |
| **Training skills which may indirectly impact on WM:**  **physical activity** | **Alesi et al. (2016)** | TD  8-9 yr. olds | Football | ✓ | RCT  Sedentary children | 44 | Yes. | Backward digit span | No significant post-intervention difference between the groups (p>.05). |
|  | **Davis et al. (2007)** | O  9 yr. olds | Physical activity | ✓ | RCT  Low dose; high dose; No intervention control. | 94 | No. | CAS successive scale | No significant post intervention group differences (p=.08) |
|  | **Kamijo et al. (2011)** | TD | Physical activity | ✓ | RCT  Waitlist | 43 | Yes | Modified Sternberg task | Effects on the 3 letter condition (not on 1 and 5 letter condition) |
|  | **Koutsandréou et al. (2016)** | TD | Physical activity | ✓ | RCT  Cardiovascular group; motor demanding group; No-exercise control. | 71 | Yes.  Specific effects for each group | Letter digit span | Pre-post intervention gains observed for both intervention groups but not for the control gp: CE *p<*.001; ME *p<*.001  Control *p=*0.391 |
|  | **Van der Niet et al. (2016)** | TD | Physical activity | ✓ | QE  Normal school routine | 112 | No | Backward digit span | Post-intervention group comparisons: significant differences between exp. and control (*p=*.02) |
| **Training skills which may indirectly impact on WM: inhibition** | **Volckaert and Noël (2015)** | TD | Inhibition | ✓ | RCT  Handicraft lessons | 47 | Yes | Categospan | *WM measure: significant pre-post intervention gains for exp. group (*p<*.001). No significant gains for control group – (*p=*.215). |

*Note: C= control group (s); E = experimental group; TD = typically developing; DS= Down’s syndrome; SLI = specific language impairment; O = overweight; LD = at risk of learning difficulties; RD = reading difficulties’ R+WM=reading and WM difficulties. QE= Quasi-experimental; RCT = Randomised Controlled Trial. BDS= backward digit span*

* Results across WM measures combined into a factor analysis so specific effects on VSSTM cannot be distinguished.

**Supplementary Material: Table B4: Synthesized findings: visuospatial ELWM**

| **Intervention Type** | **Study author and year** | **Population** | **Intervention** | | **Comparison** | | **OUTCOMES** | | |
| --- | --- | --- | --- | --- | --- | --- | --- | --- | --- |
|  |  | **status**  **age** | **Trained**  **skill** | **executive-loaded?** | **Study design**  **and comparison** | **No of participants** | **Effect on trained skill** | **VSELWM**  **outcome(s) measured** | **Effect** |
| **Adapting the**  **environment** | **Elliott et al. (2010)** | TD  5- 6 yr. olds;  9- 10 yr. olds. | None | N/A | QE  Behavioural teaching;  No intervention | 256 | No evidence of enhance strategy-use by teachers. | Odd one out  Mr X  Spatial recall | No significant pre-post intervention differences or group differences observed (*p>*.05). |
| **Direct WM training**  **without**  **strategy instruction** | **Henry et al.**  **(2014)** | TD  7 yr. olds | Listening recall  Odd one out | ✓ | RCT  Active control | 36 | Yes | Odd one out  Counting recall | Post-intervention group comparisons: Significant differences between exp. and control on both tasks Odd one out (*p<*.001) |
|  | **Passolunghi and Costa (2016)** | TD  5yr olds | All 4 aspects of WM | ✓ | RCT  Numeracy training  No intervention | 48 | Significant effects on EWLWM but not on STM tasks. | Visuospatial dual task | Significant post-intervention group difference between exp. versus numeracy (*p=*.03); and exp. versus control (*p<*.001) |
| **Direct WM training with strategy instruction** | **Caviola et al. (2009)** | TD  8-9 yr. olds | Corsi block tapping | 🗶 | QE  General cognitive strategies | 46 | No effect on VSSTM trained task. | Backward Corsi  A near-transfer measure | Post-intervention group comparisons – non significant (*p=*.03) |
| **Training skills which may indirectly impact on WM:**  **physical activity** | **Van der Niet et al. (2016)** | TD | Physical activity | ✓ | QE  Normal school routine | 112 | No | Visual memory span | Post-intervention group comparisons - not significant (*p=*.115) |

**Supplementary Material: Table B5: Synthesized findings: near- and far - transfer effects.**

| **Intervention Type** | **Study author and year** | **Population** | **Intervention** | | **Comparison** | | **NEAR-TRansfer EFFECTS** | | **FAR-TRANSFER EFFECTS** | |
| --- | --- | --- | --- | --- | --- | --- | --- | --- | --- | --- |
|  |  | **status**  **age** | **Trained**  **skill** | **executive-loaded?** | **Study design**  **and comparison** | **Total sample** | **outcome(s) measured** | **Effect** | **outcome(s) measured** | **Effect** |
| **Adapting the environment** | **Elliott et al.**  **(2010)** | 5- 6 year olds  9-10 year olds | No directly trained task | 🗶 | QE  Behavioural teaching;  No intervention | 256 | N/A due to nature of the intervention | - | 5- 6yr olds cohort – vocabulary  9- 10 yr. old cohort –reading and maths | No significant effects found |
| **Direct WM training**  **without**  **strategy instruction** | **Henry et al.**  **(2014)** | TD  7 yr. olds | Listening recall (VELWM)  Odd one out  (VSELWM) | ✓ | RCT  Active control | 36 | Digit recall (VSTM) | Group comparisons on post intervention- No | Number skills  Spelling  Reading comprehension | No significant effects found except on reading comprehension task which was introduced at 12 month follow-up (p<.01). |
|  |  |  |  |  |  |  | Word recall (VSTM) | Group comparisons on post intervention- Yes. *p<*.01 |  |  |
|  |  |  |  |  |  |  | Counting recall (VELWM) | Group comparisons on post intervention- Yes. p,.05 |  |  |
|  |  |  |  |  |  |  | Block recall (VSSTM) | Group comparisons on post intervention- No. |  |  |
|  | **Banales et al. (2015)** | R+WM  9 year olds | N-back  Listening recall | ✓ | Case series  Each child own matched control | 4 | Not measured | - | Reading: sight word recognition and word decoding | No effect |
|  | **Passolunghi and Costa (2016)** | TD  5yr olds | All 4 aspects of WM | ✓ | RCT  Numeracy training  No intervention | 48 | Not measured | - | Numeracy | WM training group and numeracy groups made greater gains than the control group but there was no difference between the two intervention groups. |
| **Direct WM training with strategy instruction** | **Caviola et al. (2009)** | TD  8-9 yr. olds | Corsi block tapping (VSSTM) | 🗶 | QE  General cognitive strategies | 46 | Forward digit span | No. *p=*.62 | Not measured | - |
|  |  |  |  |  |  |  | Backward digit | No. *p=*.10 |  |  |
|  |  |  |  |  |  |  | Visual patterns | No. *p=* .27 |  |  |
|  |  |  |  |  |  |  | Backward Corsi | Yes. *P=*.03 |  |  |
|  | **Cornoldi et al. (2015)** | TD  9 yr. olds | Word list updating | ✓ | QE  Cross over design-  Usual maths lessons | 135 | Not measured | - | Arithmetical problem solving | both groups improved during trained periods and not during the untrained periods.  Regression analyses indicated that the only significant predictor of problem solving was a gain in WM updating. |
|  | **Peng and Fuchs (2015)** | LD  7 yr. olds | Counting recall  (VELWM) | ✓ | RCT  Without strategy  No intervention | 58 | Digit recall (VSTM) | No effect | Listening comprehension and retell. | On the QRI Retell measure the strategy group significantly outperformed the control group whereas the no-strategy group did not  On the QRI listening comprehension measure, both intervention groups outperformed the control condition.  Strategy use: children in the strategy instruction group were observed using rehearsal on 89% of trials compared to 17% for the no-strategy group. |
|  |  |  |  |  |  |  | Block recall (VSSTM) | No effect |  |  |
|  |  |  |  |  |  |  | Listening recall (VELWM) | No effect |  |  |
|  | **Witt (2011)** | TD  9 yr. olds | Backward digit span, updating and counting recall  (VELWM) | ✓ | QE  No intervention | 38 | Visual patterns (VSSTM) | Significant post-intervention group difference (*p<*.05) | Maths addition time and accuracy | Significant effects on addition time.  No change on children’s addition time. |
| **Training skills which may indirectly impact on WM: inhibition** | **Volckaert and Noël (2015)** | TD | Inhibition | ? ✓ | RCT  Handicraft lessons | 47 | N/A due to nature of the intervention | - |  | UCG - experimental group showed significantly less negative behaviours after training. Control group showed more. Conners scale - parents reported no effects on conduct or impulsivity scale but significant effects on hyperactivity. Teachers (blinded to the children’s group) did not report improvements on conduct or hyperactivity scales. Significant improvement on inattention scale for the experimental group but not for the control group. |
